# Dynamical changes of land use/land cover and their impacts on ecological quality during China’s reform periods: A case study of Quanzhou city, China

**DOI:** 10.1371/journal.pone.0278667

**Published:** 2022-12-13

**Authors:** Weihua Pan, Shuiying Wang, Yan Wang, Yongjiang Yu, Yanyan Luo

**Affiliations:** 1 Fujian Key Laboratory of Severe Weather, Fuzhou, Fujian, China; 2 Institute of Meteorological Science of Fujian Province, Fuzhou, Fujian, China; 3 College of Environment and Resources, Fuzhou University, Fuzhou, Fujian, China; 4 School of Tourish, Huangshan University, Huangshan, Anhui, China; 5 Fujian Meteorological Publicity Science Education Center, Fuzhou, Fuijan, China; 6 Putian Meteorological Bureau, Putian, Fujian, China; Northeastern University (Shenyang China), CHINA

## Abstract

The rapid growth of China’s economy has greatly accelerated the process of urbanization during China’s reform periods. Urbanization has significantly caused land use and land cover (LULC) changes and thus has impacts on the local climate and ecosystem. This study chooses Quanzhou, a fast-developing city of southeast China, as an example to detect and quantify the LULC and ecological changes from 1989 to 2018 by using the remotely sensed technique. The LULC of Quanzhou was derived from the four Landsat images taken in 1989, 1999, 2007 and 2018, and the land-use-degree ratio index and land-use–change method were used to estimate the change of land use. The remote sensing based ecological index (RSEI) was used to detect the ecological changes of the city. The built-up land expansion intensity and annual built-up land expansion rate were carried out for seven districts of Quanzhou. The results show that the urban area of Quanzhou has drastically grown by 192.99 km^2^ at the expense of forest, water, and cropland land during the 1989~2018 period. Moreover, the built-up land of seven districts had expanded at the average rate of 0.027~0.154 per year and the built-up expansion intensity was higher than 0.59. The average RSEI value of Quanzhou city dropped from 0.78 in 1989 to 0.34 in 2018, which suggested an overall decline in ecological quality. The proportion of areas with an RSEI rating good decreased from 30.84% to 11.52% while the proportion of areas with rating bad increased from 4.73% to 19.11% during the past 29 years. This study has shown the built-up land expansion intensity is negatively correlated with the ecological quality change, and the increase in built-up land can greatly accelerate the decline of the ecological quality. Government policies play a profound impact on land use changes, urbanization and eco-environment changes. Therefore, the policy decision-makers should take enough action and consider integrating the concept of ecology to enable the healthy and sustainable development of the city.

## Introduction

Urbanization is a major event in China during the reform periods, which has altered the natural landscape and resulted in a series of climatic and social problems. Urban growth and sprawl have drastically modified the biophysical environment [1–3]. The noteworthy is the replacement of soil and vegetation with impervious urban materials, such as concrete, asphalt, and buildings, thus significantly impacting the land-atmosphere energy exchange processes, and several noticeable phenomena that have arisen as a result of urban expansion are that urban climates are warmer and more polluted than the rural counterparts and more and more land is deserted rapidly short of precipitation. And urban climate, land use and population distribution pattern have very close and complex relationships [4–7]. From broader perspective urbanization is one of many ways in which humans are altering the land cover of the globe, and the changes are estimated to have significantly altered more than 80% of the Earth’s land area over the last several centuries [8, 9]. The process of urbanization has changed the original natural ecological environment, resulting in great changes in land use and land cover (LULC). The LULC research has gradually become one of the important impetus in the relationship between human activities and ecological environment [10–12]. On the one hand, land use change, especially the process of replacing the natural landscape pattern mainly consisting of vegetation and soil by the urban landscape pattern mainly consisting of artificial buildings, has resulted in a series of ecological environment problems, such as air pollution, soil degradation, urban heat island effect and reduction of biodiversity. On the other hand, with the increasing awareness and attention of human society to the above environmental problems, managers and decision makers will adjust land ultimately strategies independently according to the changes of ecological environment and ultimately affect land use change, which can be regarded as the restriction or influence of ecological environment on land use patterns.

In the context of rapid urbanization, It could make the population and industrial activities from scattered to agglomerated, and more likely to produce the heat island effect during the process of urbanization, which has a huge impact on the regional ecological quality [13, 14]. LULC change detection provides a fundamental input for planning, management and environmental studies, such as landscape dynamics or natural risks and impacts [15–17]. Of critical importance is linking these observed changes in built-up land to the driving socio-economic or environmental origins [18, 19]. In particular, the LULC change offers a graphic depiction of the interplay between economics, political systems, and the environment [20–22]. One technology that offers considerable promise for monitoring LULC change is satellite remote sensing, because of its temporal resolution, provides an excellent historical framework for estimating the spatial extent of LULC change and a repetitive measurement of earth surface conditions relevant to climatology, hydrology, oceanography and land cover monitoring [23–25]. Recent advances in remote sensing technology have provided a large amount of land surface data for ecosystem monitoring and a robust indication of ecological conditions at different scales [26–29]. some remote sensing indices have been created to quantify ecological status [30–33]. Some authors assessed the sensitivity of several spectral indices to land surface moisture conditions and found the temperature/vegetation index had the highest sensitivity to moisture intensity [34]. Other authors developed techniques of land surface temperature (LST) and vegetation for urban planning assessment [35]. The study showed that LST retrieved from remote sensing thermal imagery was reliable in evaluating urban heat effects. Some researchers used the normalized difference vegetation index (NDVI) for remote monitoring of groundwater flows in the Australian Great Artesian Basin [36]. Aggregated remote sensing based ecological indices monitor ecological status with two or more metrics and thus can be used in the identification of more features related to ecological status. Some authors combined remote sensing metrics with GIS to analyze the spatio-temporal pattern of urban ecological security [37]. Other authors used three indicators (hyperspectral remote sensing data, GIS derivation and height information) to develop urban ecological indicators [38]. Scholars found that the growing season of vegetation in urban areas was significantly different from that in rural areas, which had generally been attributed to the influence of the urban heat island effect on vegetation phenology along the urban-rural gradient [39]. Some researchers designed remote sensing based ecological index (RSEI) aggregated four indicators representing climatic and land-surface biophysical variables [40]. The results showed that the RSEI index might reveal successfully the comprehensive ecological status of the study areas and provide early warnings of future changes potentially impacting habitat suitability [41].

Many scholars continue to explore the effects of LULC and urbanization on local ecological quality. Some authors used multi-temporal SPOT images to study the influencing factors of land use change and ecological environment in Vietnam [42]. Other authors analyzed systematically the temporal and spatial patterns and new characteristics of LULC in China from 2010 to 2015 [43]. Researchers studied the influence of urban architectural form on urban land surface temperature in Shanghai and provide a reference for urban architecture planning to develop urban ecologic quality [44]. Some authors analyzed the changes of ecological environment in Jiangsu province along the Yangtze River economic belt with the multi-source satellite remote sensing technology and revealed that the ecological environment quality of the urbanization areas had declined significantly from 1999 to 2020 [45]. Some scholars found that the overall ecological quality of the Lhasa urban circle showed an improving trend with an improvement proportion of 45.98% during the period 1994 to 2017 [46].

China is in a new round of urbanization, and the increase in construction land caused by urbanization is bound to put further pressure on the originally fragile ecological environment [47]. Previous studies on regional ecological environment monitoring have mostly focused on areas that had experienced rapid urbanization, but it is not clear whether the different land use conditions, different economic structures, and different urbanization processes could produce different regional ecological environment impact patterns. Since 1989, Quanzhou has experienced a rapid process of urbanization, which has led to great changes in LULC, especially the rapid expansion of built-up land. Its location in the Southern Fujian Delta area on the southeast coast of China with many mountains and few plains, and the developed local economic structure of private enterprises make it a useful representative case study for studying the regional ecological environment impact of LULC changes during the urbanization process. In this paper, Quanzhou, China, one of the most developed cities for private enterprises in China, is selected as the case study. By using Landsat remote sensing image data collected from 1989 to 2018, the LULC and ecological quality characteristic parameters of the four phases are obtained, on the basis of which the expansion intensity and expansion rate, the ecological environment of Quanzhou city and the characteristics of its spatiotemporal evolution are illustrated, and the driving factors and changes of the urban ecological quality pattern under different spatial conditions are further explored. The research results will provide scientific and technological support for similar cities around the world for local government agencies to improve the urban ecological environment and maintain a long and sustainable development.

## Materials and methods

### 2.1 Study area

**Fig 1:**
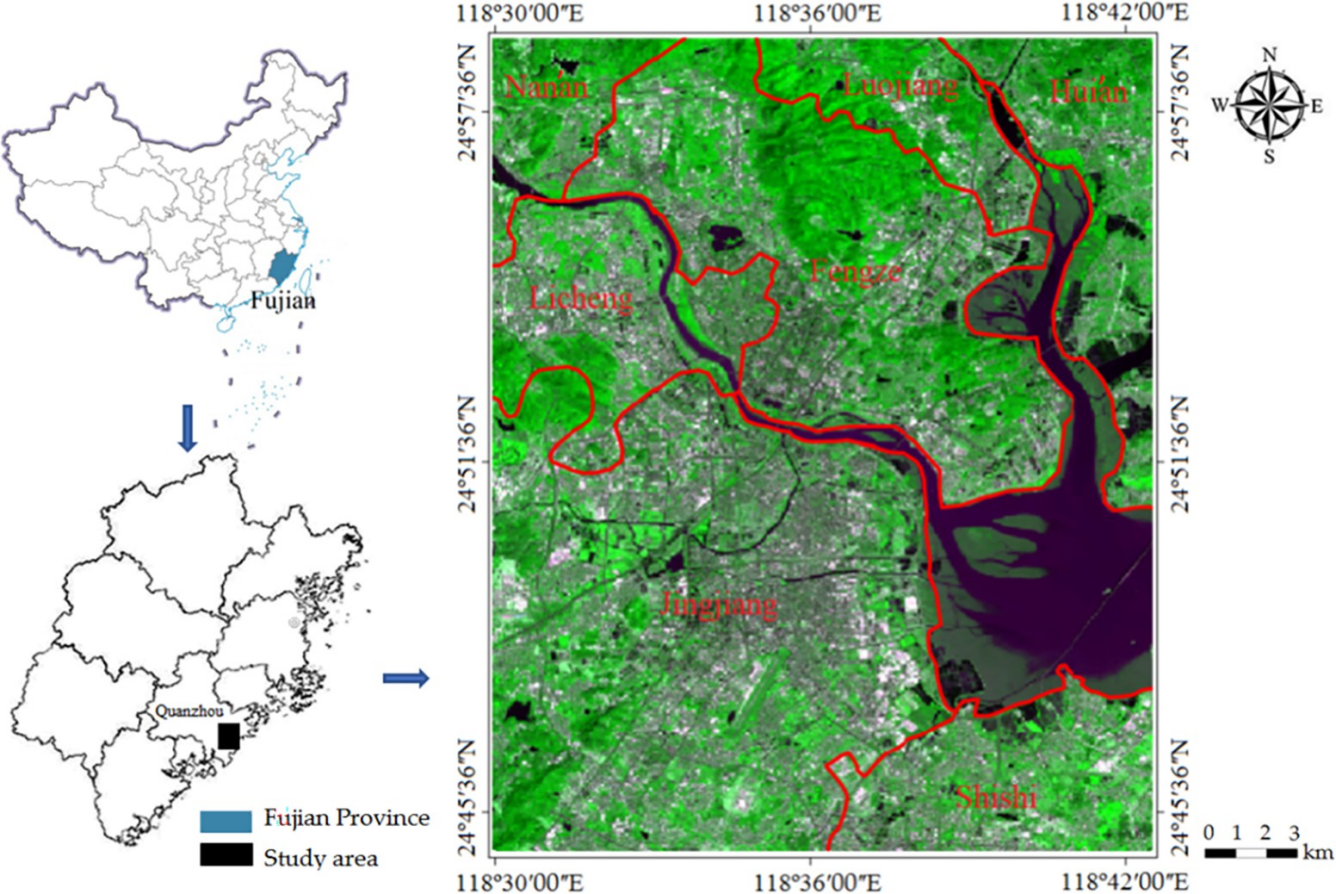
Location of the study area (Quanzhou city) (right) in the context of Fujian province (bottom left) and Fujian province in the context of China (above left).

The study area is the core area of Quanzhou city (118°30ʹ -118°42ʹ E, 24°44ʹ -24°59ʹ N) located in the coastal zonal area of Southeast Fujian province, PRC (Fig 1), including seven districts such as Licheng, Fengze, Luojiang, Jinjiang, Shishi, Hui’an and Nan’an. Quanzhou is adjacent to the Taiwan Strait and belongs to a subtropical maritime monsoon climate. The annual average temperature is 19.5~21.0°C, the annual average sunshine hours are 1900~2130 h, and the annual average precipitation is 1000~1400 mm. The topography comprises a complex landform of low hills, mountains, plains and rivers.

Quanzhou city is one of three cities comprising the famous Golden Triangle Area of Southern Fujian. Moreover, the urbanization process of Quanzhou city has stupendously developed during the reform period from 1989 to 2018, and the GDP of Quanzhou city is top in the rank of those of all cities in Fujian Province.

### 2.2 Data source and pre-processing

To generate and monitor the LULC and estimate the ecological change influenced by urbanization, four satellite images of Landsat TM and Landsat 8 OLI/TIRS for 1989, 1999, 2007 and 2018 were acquired from the United States Geological Survey (USGS) (http://earthexplorer.usgs.gov/). The cloud coverage in each image was below 5%. Both scenes were imaged during the April-June regional growing season to minimize seasonal variability. Spring or earlier summer, when vegetation was in the stage of vigorous growth, was the preferred season for the Landsat scenes. Other data sets used in this study included differential global positioning system data, land-use vector data from Fujian Administration of Surveying Mapping and Geo-information, and socioeconomic data from Quanzhou Statistical Bureau.

The images were registered in a NAD27 projection and used manual selection of ground control points (GCPs) followed by a linear (affine) transformation using nearest neighbor resampling. RMS errors for the GCP sets were less than 0.50 pixels in all cases. Considering the characteristics of the study areas, the classification was performed by means of a hybrid classifier [48].

### 2.3 Methods

**Fig 2:**
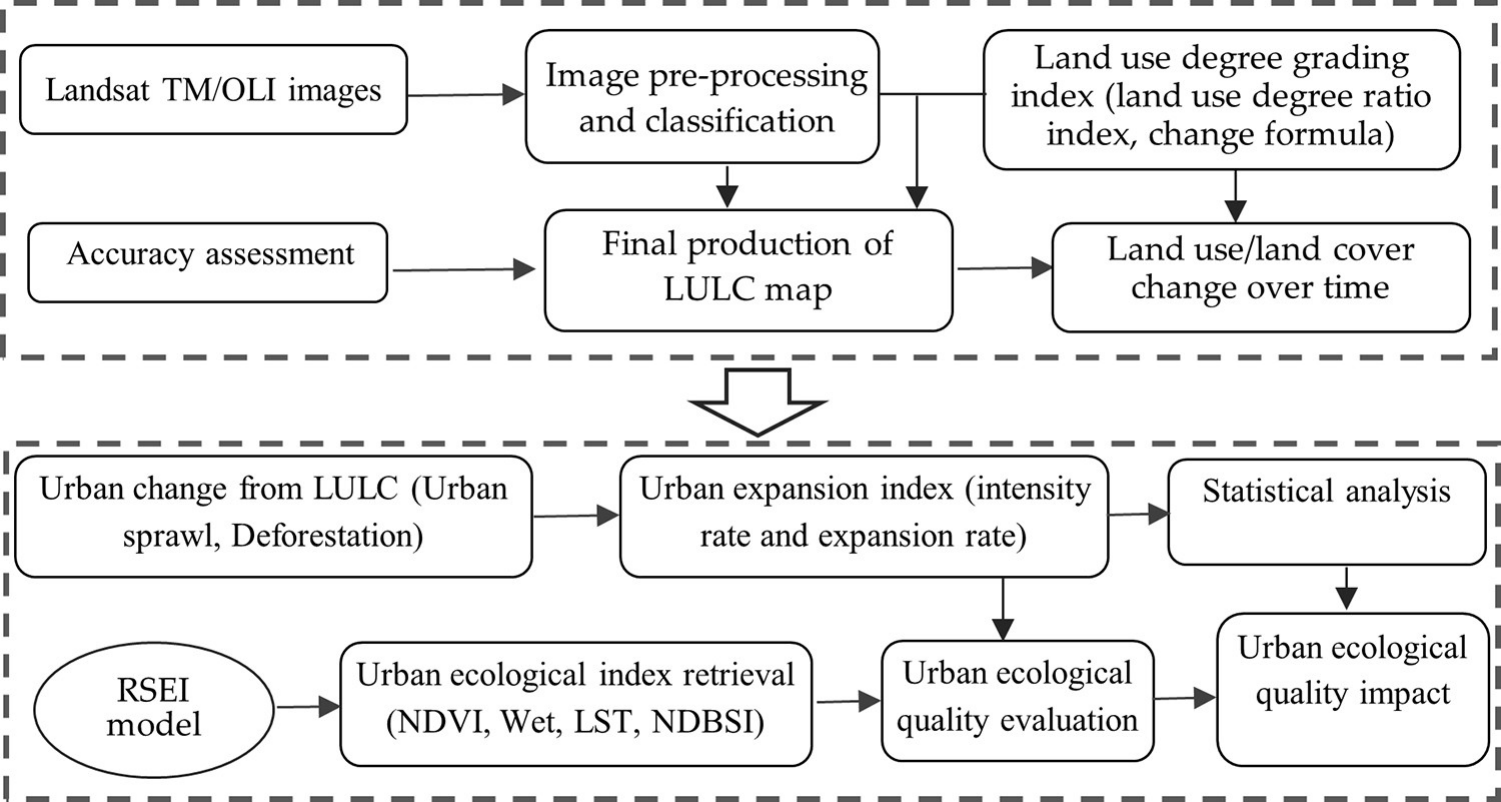
Overall methodology.

This study proposes a novel hybrid land use degree grading index, urban expansion index and RSEI model for urban ecological evaluation. Fig 2 illustrates the overall methodology of the study. It contains two interrelated major sections. The first section of the overall methodology analyzes the dynamic changes of land use/land cover change by using the land use degree grading index to estimate built-up land change. The second section details the use of the urban expansion index and the RSEI model in urban ecological evaluation. This RSEI model is selected to compare the ecological conditions in different periods. The overall methodology is described in the following two subcategories.

#### 2.3.1 Land-use-degree ratio and land-use-change index calculation

The computations of the land-use-degree ratio index and land-use–change index formula are based on the following [49]:

$$L=100\times \sum_{i=1}^{n}A_{i}\times C_{i}$$ (1)

$$R=\frac{\sum_{i=1}^{n}(A_{i}\times C_{i,b})-\sum_{i=1}^{n}\left(A_{i}\times C_{i,a}\right)}{\sum_{i=1}^{n}\left(A_{i}\times C_{i,a}\right)}$$ (2)

Where *L* is land-use-change index, *R* is land-use-degree ratio index, *A*_*i*_ is the level of land-use type, *C*_*i*_ is the percentage of the corresponding levels in the total study areas, *C*_*i*,*a*_ and *C*_*i*,*b*_ are the percentage of the corresponding levels in the total study areas in the year of *a* and *b*, respectively.

#### 2.3.2 Built-up land expansion rate and intensity calculation

The average annual expansion rate and expansion intensity of built-up land can be used to measure the expansion process of urbanization. The calculation formulas of two indexes are as followed [50]:

$$v_{i,t∼(t+n)}=(A_{i,t+n}-A_{i,t})\times n^{-1}$$ (3)

$$G_{i,t∼(t+n)}=(A_{i,t+n}-A_{i,t})\times {TA}_{i}^{-1}\times n^{-1}\times 100$$ (4)

where v_i,t~(t+n)_ is the average annual built-up land expansion rate of space unit i in the period of t~(t+n), G_i,t~(t+n)_ is the built-up land expansion intensity of space unit i in the period of t~(t+n), A_i,t+n_ is built-up areas of space unit i in the year of (t+n), A_i,t_ is built-up areas of space unit i in the year of t, TA_i_ is total area of study area, n is the number of the study years.

#### 2.3.3 Remote sensing based ecological index analysis

The remote sensing based ecological index (RSEI) [51] is composed of four indicators of greenness (NDVI), humidity (Wet), heat (LST) and dryness (NDBSI) to comprehensively reflect the regional ecological environment, which is quite comparable with the ecological environment status index (EI) of the Ministry of Environmental Protection of China. The calculation formulas of RSEI are as followed:

Moisture is calculated by the wet component of a Tasseled Cap Transformation to represent soil moisture as the component is designed to understand important attributes of soil and plant moisture [52]. The wet component of Landsat data can be calculated as [53, 54]:

$$Wet=c_{1}\rho_{1}+c_{2}\rho_{2}+c_{3}\rho_{3}+c_{4}\rho_{4}+c_{5}\rho_{5}+c_{6}\rho_{6}$$ (5)

where *ρ*_1_ to *ρ*_6_ denote bands 1 to 6 corresponding to the blue, green, red, the near-infrared (NIR), the mid-infrared (MIR1), and MIR2 bands of the Landsat image, *c*_1_ to *c*_6_ are the coefficients of the band.

Greenness is denoted with NDVI, which is the most commonly used vegetation index in measuring vegetation productivity due to its simplicity and robustness and thus has long been used as a dominant ecosystem proxy variable [55]. The NDVI is expressed as [56]:

$$NDVI=(\rho_{4}-\rho_{3})/(\rho_{4}+\rho_{3})$$ (6)

where *ρ*_3_ and *ρ*_4_ are red and NIR bands of the Landsat image, respectively [57–59].

Heat is represented by LST, which retrieves from Landsat thermal sensor data [60]. LST is an important metric frequently used to investigate ecological processes and climate change [61–63], as well as to study drought, evapotranspiration, vegetation density and surface energy balance [54, 64, 65]. The LST of Landsat 5 TM is evaluated as [66]:

$$L_{\lambda }=\mathrm{gain}∙Q_{\lambda }+\mathrm{bias}$$ (7)

$$T_{\lambda }=K_{2}/\ln(K_{1}/L_{\lambda }+1)$$ (8)

$$LST=T_{\lambda }/[1+(\lambda T_{\lambda }/\rho )\ln\varepsilon ]$$ (9)

where L_λ_ is the top of atmospheric spectral radiance W/(m^2^·sr·μm), gain is band-specific multiplicative rescaling factor from the metadata, Q_λ_ is quantized and calibrated standard product pixel values (DN), bias is the band-specific additive rescaling factor from metadata, *T*_*λ*_ is the brightness temperature, *K*_*1*_ and *K*_*2*_ are the thermal conversion constants, *λ* is the wave length of emitted radiance, *ρ* is the constant (1.438×10^−2^ mK), and *ε* is the land surface emissivity.

To Landsat 8 TIRS, the LST is calculated as followed [67]:

$$L_{\lambda }=M_{L}Q_{\lambda }+A_{L}$$ (10)

$$LST=\gamma [\varepsilon^{-1}(\varphi_{1}L_{\lambda }+\varphi_{2})+\varphi_{3}]+\delta$$ (11)

where *M*_*L*_ and *A*_*L*_ are multiplicative rescaling factor and additive rescaling factor respectively, *γ* and *δ* are the two constants of Planck, *φ*_1_, *φ*_2_ and *φ*_3_ are the parameters of atmospheric water vapor.

Dryness is referred to as built-induced land-surface desiccation and thus can be represented by the Normalized Differential Building-Soil Index (NDBSI), which is expressed by the index-based built-up index (IBI) [68] and soil index (SI) [69]. The ecological status is affected greatly by human activities. The negative result is the conversion of natural landscapes to impervious built land and thus causes the dryness of land surface [70, 71]. The NDBSI is expressed as:

NDBSI=(SI+IBI)/2 (12)

$$IBI=\frac{2\rho_{5}/(\rho_{5}+\rho_{4})-[\rho_{4}/(\rho_{4}+\rho_{3})+\rho_{2}/(\rho_{2}+\rho_{5})]}{2\rho_{5}/(\rho_{5}+\rho_{4})+[\rho_{4}/(\rho_{4}+\rho_{3})+\rho_{2}/(\rho_{2}+\rho_{5})]}$$ (13)

$$SI=[\left(\rho_{5}+\rho_{3})-(\rho_{4}+\rho_{1}\right)]/[\left(\rho_{5}+\rho_{3})+(\rho_{4}+\rho_{1}\right)]$$ (14)

where *ρ*_1_, *ρ*_2_, *ρ*_3_, *ρ*_4_ and *ρ*_5_ represent the blue, green, red, the near-infrared (NIR), and the mid-infrared (MIR1) bands of the Landsat image, respectively.

Instead of using a traditional weighted sum approach, this study utilized a principal component analysis (PCA) method to combine the four metrics to form RSEI and selected the first component of PCA (PC1) to represent RSEI because PC1 explicates more than 76% of the total variation of the dataset [72]. Accordingly, initial *RSEI*_0_, *RSEI* is represented by PC1 [47]:

$$RSEI_{0}=1-\{PC1[Wet,NDVI,LST,NDBSI]\}$$ (15)

The values of the four metrics should be normalized between 0 and 1 before the performance of PCA because the data range and unit of the four metrics are different.


$$RSEI=(RSEI_{0}-RSEI_{0\min})/(RSEI_{0\max}-RSEI_{0\min})$$ (16)

RSEI was further normalized between 0 and 1. This procedure puts the scores for RSEI on a common scale between 0 and 1 for easy comparison with 1 denoting perfect ecological status and 0 indicating extremely poor one.

## Results

### 3.1 LULC change analysis

For this study, a common legend is established based on the official census and actual land cover characters of Quanzhou city. In the cases of 1999, 2007 and 2018, nine LULC categories are added to the image classification scheme: paddy field, dry land, forest, grass, orchard land, built-up land (urban and village, etc.), unused land, beach, water (river and ocean). For 1989 the same classes as in 2018 are used plus two new categories: cloud and shadow because of air conditions.

**Fig 3:**
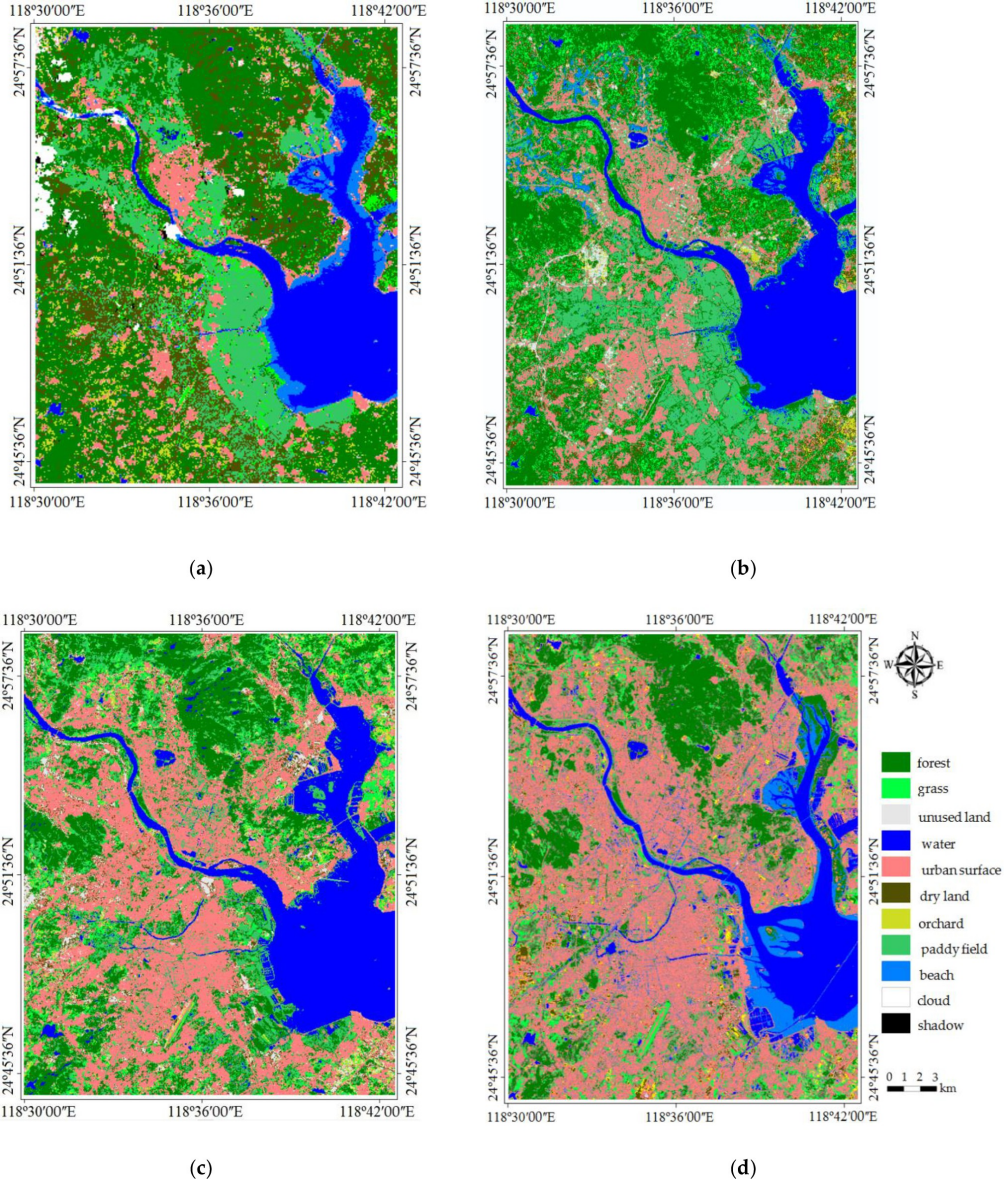
Result of supervised classification of Quzhou. (a): 1989. (b): 1999. (c): 2007. (d): 2018.

Once the four LULC classification maps (1989, 1999, 2007 and 2018) have been obtained, a major problem is detected, apparently caused not by the classification method but by the inherent problem of spectral confusion, such the phenomenon as the homogeneous objects have different spectrum values but the heterogeneous objects have the same spectrum values in images. The problem is the appearance of areas erroneously classified as arable land instead of orchard land, urban surfaces instead of dry land, and so on. This is a consequence of the confusion between different land cover spectrums, perhaps because they have a very similar radiometric response due to seasonal differences. The solution is to visual examination of classification maps with the help of investigation in field, digital topography maps, referenced ground data and unsupervised classification maps. Finally, the boundary errors caused by spectral mixing as well as spectral confusion due to the similarity of spectral signatures of several land use and land cover classes have all been corrected and the resultant classification maps are shown in Fig 3.

Accuracy assessments are performed for 1989, 1999, 2007 and 2018 LULC classification maps. Based on stratified random samples of 2364, 2468 and 2512 pixels selected from the 1989, 1999 and 2007 maps, an overall accuracy of 85.02, 86.56 and 87.82 percent are obtained. In terms of producer’s and user’s accuracies, a minimum of 80 percent is reached for eight classes (except that the accuracy of dry land is 79.41 percent). As for the 2018 land use/land cover map produced, a stratified random sample of 2344 pixels revealed an overall accuracy of 86.86 percent. Both producer’s and user’s accuracies are over 80 percent (except that the accuracy of dry land is 79.22 percent in 1989). Therefore, the four land use and land cover maps are comparable in accuracy despite differences in the type of Landsat images used (the 1989s and 1999s images are acquired by Landsat 5, the 2007s image is acquired by Landsat 7, and the 2018s image is acquired by Landsat 8).

**Fig 4:**
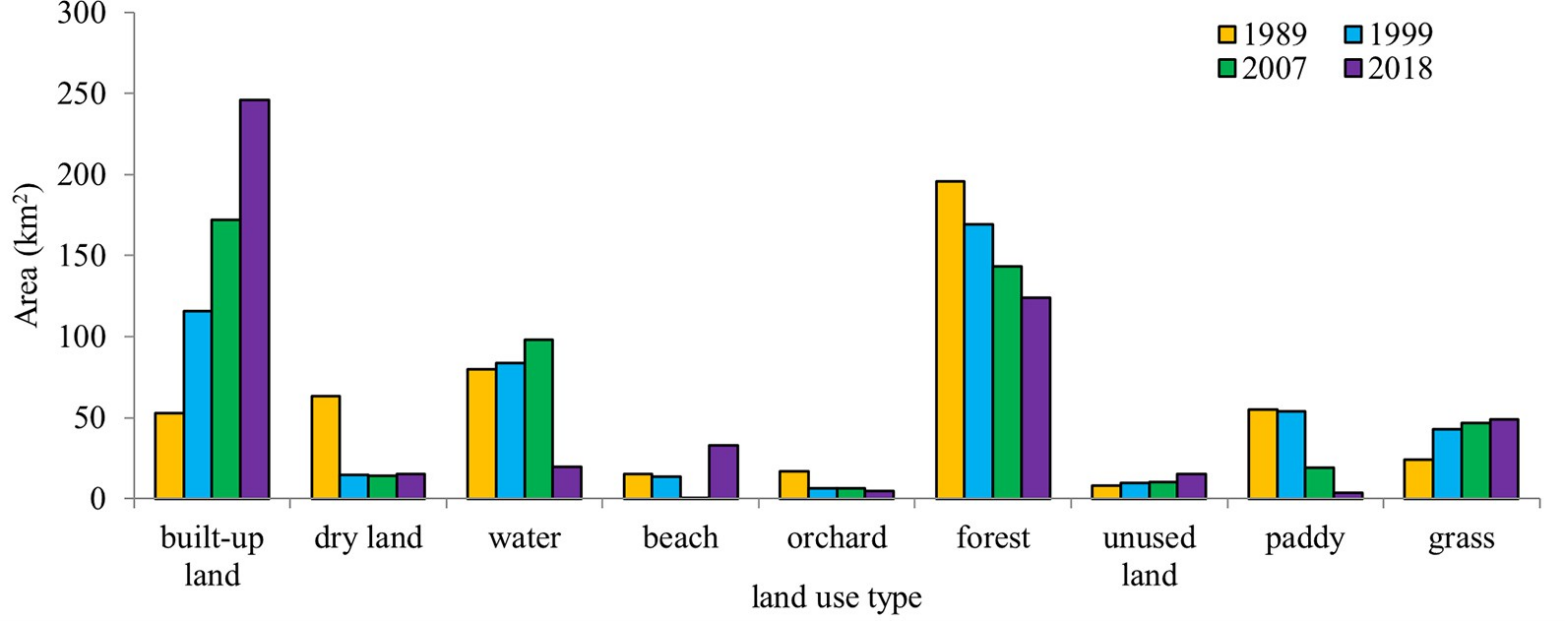
Area of land use type of Quanzhou during 1989–2018.

The area of land-use types in each of the four study images had been obtained and regional characterizations of LULC changes were understood for Quanzhou city over the 29 years from 1989 to 2018 (Fig 4). The data revealed that substantial change took place during the 29 study years. The area of paddy, dry land, orchard and forest decreased, the arable land reduced the most, while the area of grass, unused land and built-up lands such as settlements and industrial land, and transportation land increased from 1989 to 2018. The most notable was that the built-up land had increased sharply in Quanzhou city. It occupied 52.83 km^2^ of land in 1989 and increased to 245.82 km^2^ in 2018, an increase of 192.99 km^2^ in 29 years. The increase rate was 5.45% annually. The high-speed increase in the built-up land area indicated that the newly cleared area for construction in 2018 was much more than those in 1989. The area of grassland and unused land had increased by 24.44 km^2^ and 7.46 km^2^, respectively. Meanwhile, the arable land decreased from 135.24 km^2^ in 1989 to 24.02 km^2^ in 2018, and the area of paddy decreased the most among them, with a decrease of 51.21 km^2^, followed by those of dry land and orchard land. And the area of forest land decreased from 195.62 km^2^ in 1989 to 124.21 km^2^ in 2018, a decrease of 71.41 km^2^. Within the 29 years, the areas of built-up land and grassland increased steadily, and the areas of forest land, arable land, and orchard land decreased continuously. Therefore, the areas of water bodies increased first and then decreased.

From Fig 3, the numerous formal arable lands had been occupied by residential complexes, indicating that agricultural soil was lost to the built-up land class. The arable land was lost 111.22 km^2^ in the 29 years, of which most was lost to built-up land use. The loss of agricultural fields to urban land could be examined especially in the areas of Licheng, Fengze and Jin’an counties, where many large patches of paddy fields and orchard land had transformed into residential sectors between 1989 and 2018.

**Fig 5:**
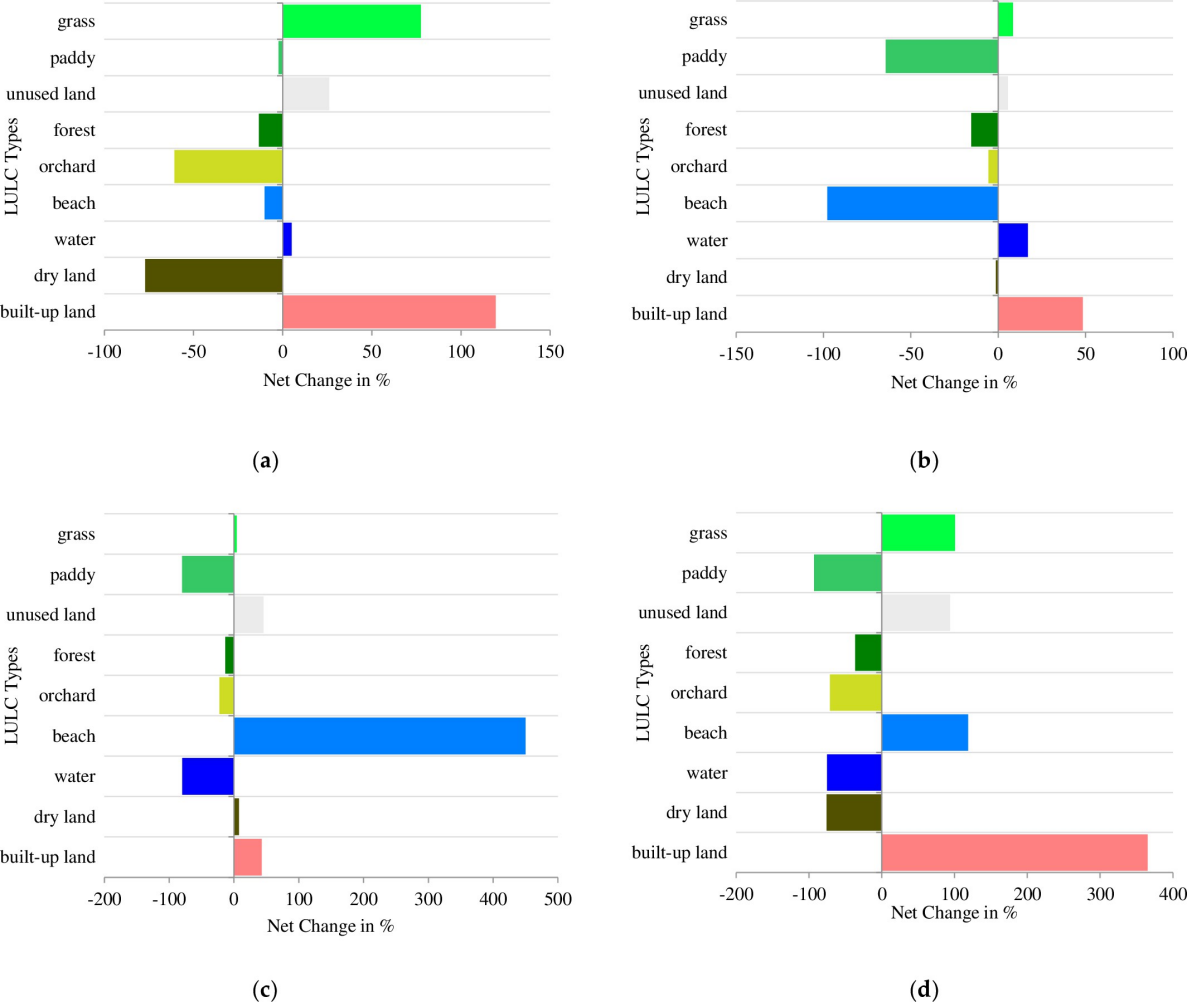
Net Changes in LULC types of Quanzhou during 1989–2018. (a): 1989–1999. (b): 1999–2007. (c): 2007–2018. (d): 1989–2018.

Therefore, the LULC change scenarios of the study area suggest the rapid development and expansion of built-up land during the reform periods. This increase in built areas may have contributed to the negative changes in some LULC classes, as illustrated in Fig 5.

From Fig 3B and 3C, it can be seen that the areas with the obvious spatial expansion of build-up land in the study area from 1999 to 2007 are mainly distributed in Chendai Town and Xibin Town in the northeastern coastal zone, Zimao Town in the west and Lingyuan Street in the middle of Jinjiang City, and Huada Street in the northeastern coastal zone of Fengze District. From Fig 3A and 3D, the forest located in relatively flat terrain in northern and western Quanzhou had undergone significant deforestation in 29 years, where 71.41 km^2^ of forests were lost from Fig 4. Fig 5A and 5D showed that lost forest and arable land were mainly converted to built-up land, especially in the plains and the base areas of mountains/hills. Some of the lost forests had transformed into grass which had also increased in the study years. From Fig 5B and 5C, the areas of beaches changed greatly in 1999 and 2007 because of the rise and fall of seawater.

Our study indicated the LULC of Quanzhou city had been developing during the past 29 years, and the urban area had grown by 192.99 km^2^. Overlaying classified images from 1989 and 2018 indicate the land cover conversion associated with the rapid urbanization development. While residential development had strongly favored the occupying of arable land, the clearing of forests and the filling of the water body. Commercial development had converted both agricultural and forested land. This may reflect consumer preference for watered homesites, or it may reflect a lack of arable land adjacent to existing communities where new residential growth is likely to occur, which caused the competition between the arable land and the construction land [73].

It is worth noting that the arable land has been in a decline in progress during the past 29 years. The total amount of arable land has decreased stupendously, mainly occupied by new residential growth. Another factor that leads to arable land reduction lies in the transformation of the people’s concept. Because of the high market value of breeding aquatics and planting fruit trees instead of planting traditional crops, such as rice, wheat and earthnut. Nevertheless, to our great pleasure, the nations have called for protecting the arable land and actualizing strict policies to forbid misusing land resources [74, 75]. Moreover, substantial parts of the urban area have been reforested, most likely as cleared agricultural landfall.

### 3.2 LULC change degree evaluation

To measure the rates of LULC change, a change analysis technique has to be applied to the four classified maps. Based on the exploitation degree of the land cover, the land-use classes have been grouped into 4 levels in this study. Level 1 is mainly distributed in the unused land because of its origin, and level 2 is mainly composed of forest, water and grassland. Accordingly, level 3 is made up of arable land, orchard land and man-made lawn, and the highest 4 level includes mainly urban surfaces, villages, mine and transportation roads, and so on.

The calculation of the *L* and *R* is on the basis of the ratio of the LULC area to the total study area with the consideration of weighted values of each land-use type level (Table 1). For evaluation, the value of *R* is bigger than the zero value means that the LULC of the study area is in the developing stage. On the contrary, it means in the adjusting or declining stage [49].

**Table 1:** Land use degree grading index table in China.

Classified grade	Unused cultivation	Extensive cultivation	Intensive cultivation	Town assemble exploitation
Land use type	Unused land	Forest, water, grass	Arable land, orchard, man-made lawn	Urban surfaces, villages, mine, traffic roads
Exploitation degree	1	2	3	4

As a result, the values of L and R in Quanzhou city during the period of 1989 to 2018 are calculated as followed in Table 2. The calculated values of L in 1989, 1999, 2007 and 2018 are 247.74, 261.52, 271.40 and 288.04, respectively, showing an overall upward trend. By comparing the values of **ΔL and R**, It can be seen that the growth in the periods of 1989–1999 and 2007–2018 are faster than that in the 1999–2007 period, meaning the changes of LULC in rapid growth stages. Although the value of **R**_**1999-2007**_ is smaller than 0.05, the change of LULC has still reached a fast level. From Table 2, we can make a judgment that the LULC of Quanzhou city is in a rapidly developing stage. The increase in values of R indicates that human activities have affected land use comprehensively. The value of R is the largest in the period of 2007 to 2018, with a value of 0.0613, indicating the social and economic activities in Quanzhou have a greater impact on land use than the periods of 1989–1999 and 1999–2007.

**Table 2:** The amount and rate of land use change in Quanzhou from 1989 to 2018.

L_1989_	L_1999_	L_2007_	L_2018_	ΔL_1989-1999_	ΔL_1999-2007_	ΔL_2007-2018_	ΔL_1989-2018_	R_1989-1999_	R_1999-2007_	R_2007-2018_	R_1989-2018_
247.74	261.52	271.40	288.04	13.78	9.88	16.64	40.30	0.0556	0.0377	0.0613	0.1627

The smart change of LULC in Quanzhou city highlights regional economic and political variations. It is evident that the economy of Quanzhou city increases at a high speed, and the GDP of the city ranks No.1 in Fujian province. At present, the levels of urbanization and industrialization are rather higher because of economic extraversion. Based on the census data of local jurisdictions, the larger amount of straight foreign investments swarm into the city and capital construction such as factories, transportation road-building appear much more pronounced than before.

The correlation between population increase and LULC change appears particularly strong for the Quanzhou area. On one hand, these quick growth rates of LULC correlate with regional population suggesting more people’s inhabitation to build, which drives the land cover to change. The change in land use will also cause a change in local climate, which affect the spatial pattern of the population. On the other hand, with the increase of personal income data, it certainly requests that the development of the region should keep pace with the major economic and developing trend. Indeed, it is fairly reliable to consider that these underlying population variations may be reflected in land cover changes [76–78].

### 3.3 Built-up land expansion rate and intensity analysis

With the rapid development in economics and further advance in reform policy, the urban area has enlarged from 52.83 km^2^ in 1989, to 245.82 km^2^ in 2018, with 5.44 rate per year on average. The capacity of urban development to radiate from sub-district centers to surrounding new towns has evidently increased, especially in Jinjiang and Shishi. With urban expansion evidencing a certain orientation, Quanzhou has developed rapidly. The extent of urban expansion is in close agreement with the orange expansion range.

**Fig 6:**
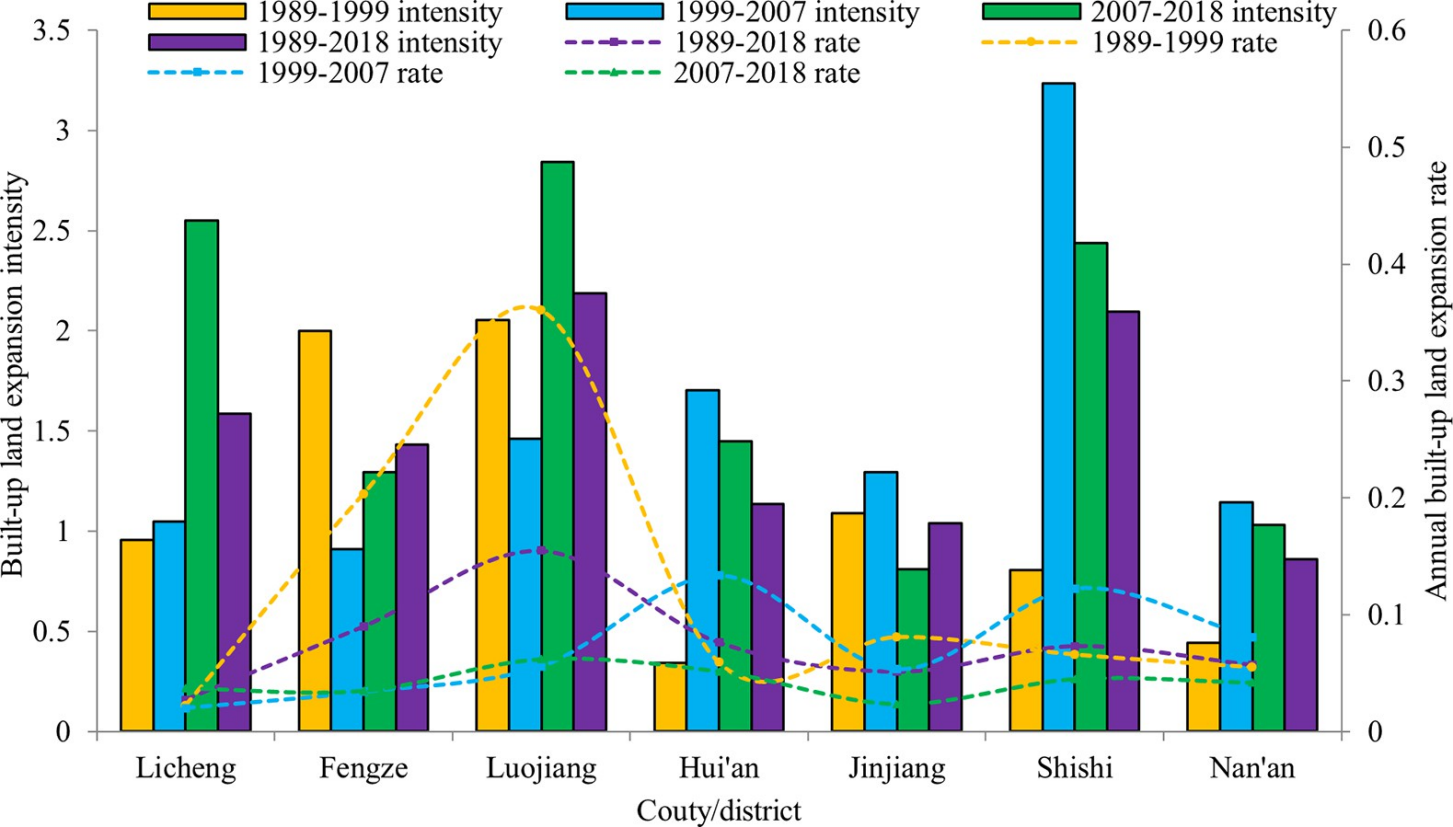
Built-up land expansion intensity and annual built-up land expansion rate showed by each County during 1989–2018.

Fig 6 shows the built-up land expansion intensity and annual built-up land expansion rate of different districts in Quanzhou. Evidently, the built-up land had been expanded at the average rate of 0.027~0.154 per year during the 1989~2018 period. It is extraordinarily high, especially in the region of Luojiang district, and that of build-up land is maintained at 0.154 per year. The built-up expansion intensity of each district was higher than 0.59, suggesting the urban expansion at the level of medium to high-speed spread [50, 79].

The built-up expansion intensity and spatial continuity are closely related to the original scale of the city [80]. With the rapid economic development, the growth centers of built-up land shift gradually from the inner to the outside, and the industrial land is the most important element of built-up land growth in Quanzhou. However, the concentric sprawl pattern seriously destroyed the spatial structure of the original land covered by forest and arable land, and also badly worsened the regional ecological environment.

### 3.4 Ecological status

#### 3.4.1 Spatio-temporal change of ecological quality

**Fig 7:**
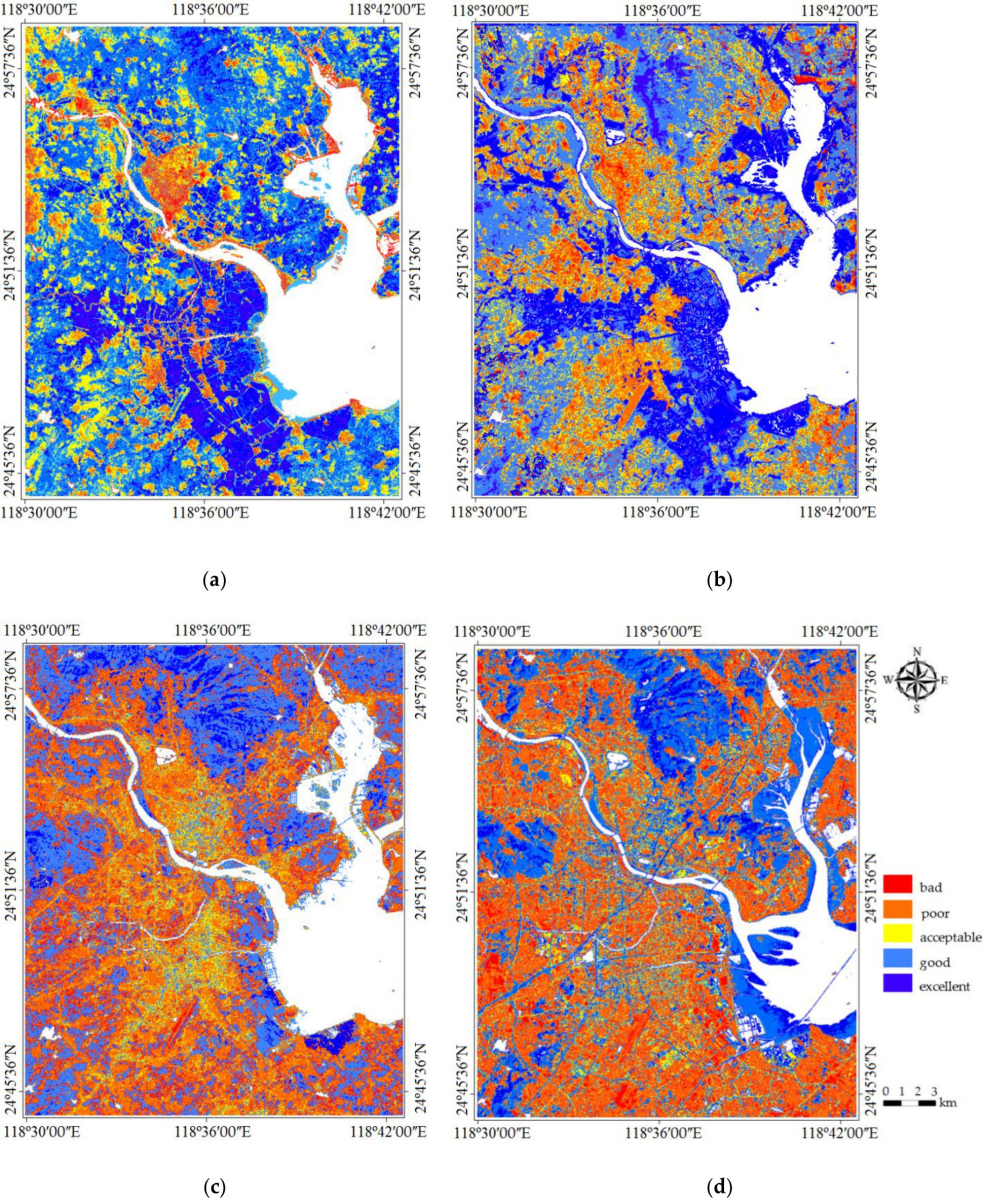
RSEI value of Quanzhou. (a): 1989. (b) 1999. (c) 2007. (d) 2018.

To compare the ecological conditions in different periods, the RSEI is subdivided into five levels at 0.2 intervals comparable with those of the Ecological Index (EI) used by the Ministry of Environmental Protection of China. The divided five levels are Level 1 (0–0.2): bad, Level 2 (0.2–0.4): poor, Level 3 (0.4–0.6): acceptable, Level 4 (0.6–0.8): good and Level 5 (0.8–1): excellent [81]. Fig 7 is the RSEI images of the study years that comprehensively illustrate the ecological conditions of the city and their spatial variations in the study period. From the RSEI images of different years, the ecological quality of Quanzhou city has significantly changed from 1989 to 2018. The bluish patches representing good to excellent ecological condition areas distribute along with topography and coastal zonal areas, which are dominated by well-forested hills and agricultural land. The reddish patches denoting poor to bad ecological conditions scatter over relatively flat lowland areas in the city, which is densely populated and intensively developed. The visible RSEI images make the index more understandable because the ecological status of a region is now characterized by numerous RSEI values. In 1989, the large red areas in Fig 7A, which represent poor ratings, were mainly located in the main urban region while the areas with red dots were distributed in the suburbs. Areas with excellent ratings were mainly located in the southern part of Quanzhou, where a large amount of agricultural land was distributed. In 2018, there was a significant decrease in areas rated as excellent compared with such areas in 1989. These results indicated that there was a great increase in areas rated as bad and poor, with a significant decrease in areas rated as excellent and good (Fig 7D). As a result, the average RSEI value of Quanzhou city dropped from 0.78 in 1989 to 0.34 in 2018, which indicated an overall decline in ecological quality.

From Fig 7A and 7B, it can be seen that the areas with low ecological quality (Level 1 and Level 2) are mainly located in the urban areas and towns of Licheng, Fengze, Jinjiang and Shishi, and the areas with good ecological quality (Leve3 and Level 4) are located in the paddy fields, orchards, forests and towns of Jinjiang and Shishi. With the rapid urbanization, the areas with low ecological quality spread rapidly and occupy most of the areas in Licheng, Fengze, Jinjiang and Shishi, and the areas with good ecological quality are mainly located in the mountains of Licheng and Fengze (Fig 7C and 7D).

**Fig 8:**
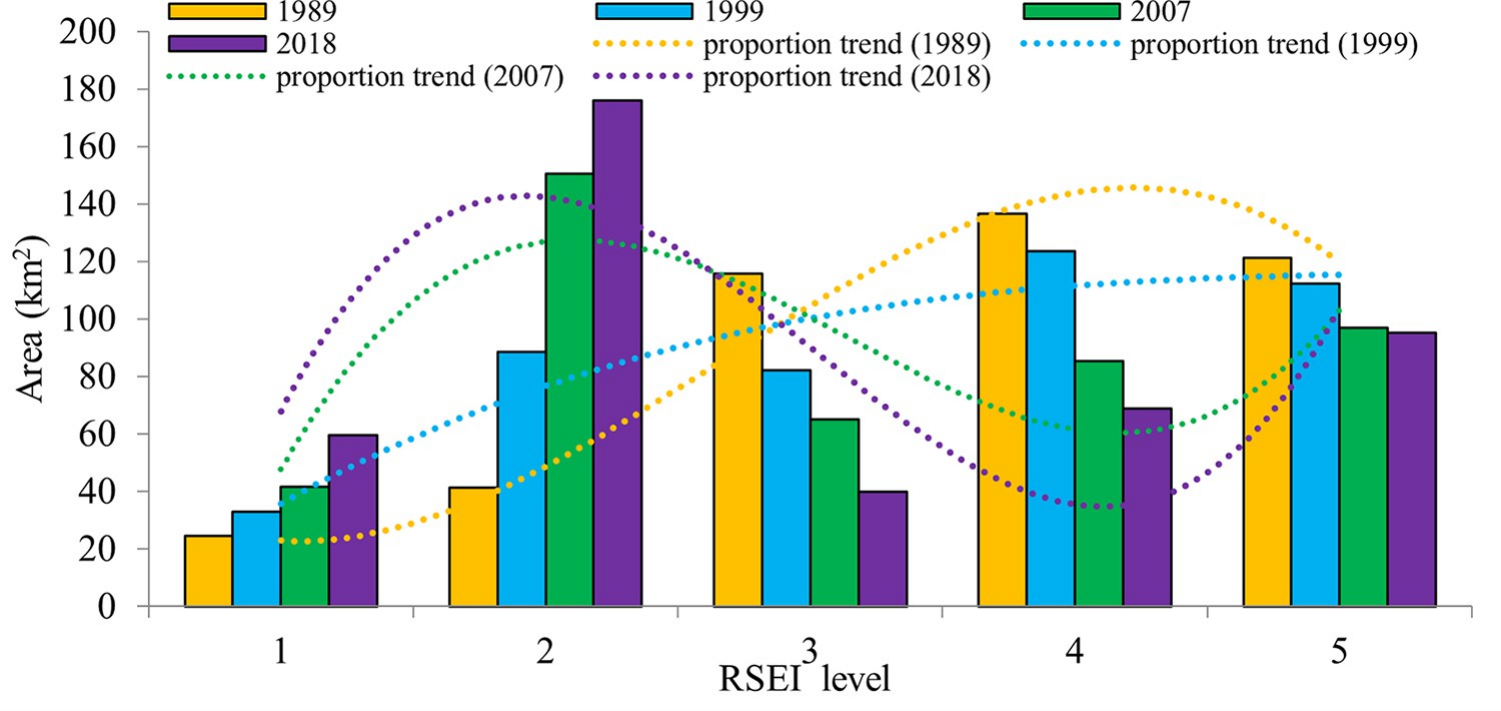
Change areas of each RSEI level from 1989 to 2018.

To develop a better understanding of the quantitative characteristics of RSEI in Quanzhou, we calculated the areas of different years according to their ratings (Fig 8). From Fig 8, the areas rated bad increased from 24.64 km^2^ in 1989 to 99.43 km^2^ in 2018, and the areas rated poor increased from 53.86 km^2^ to 195.89 km^2^ in 29 years. By contrast, the areas rated good decreased from 160.45 km^2^ to 59.92 km^2^, and the areas rated excellent from 139.53 km^2^ to 118.11 km^2^ during the study periods. Evidently, the curve reflects that the predominant RSEI rating was good in 1989, and the proportion of areas with a rating of good and excellent was 57.66%. Notably, the predominant RSEI rating in 2018 was converted to the poor, and the proportion of areas with a rating of poor and bad was 56.76%.

The overall ecological situation had significantly changed from 1989 to 2018, and the proportion of areas with a poor rating in 2018 increased to 37.56%, evidencing an increase of 27.29% compared with that in 1989. Areas rated as average decreased 94.87 km^2^, with their proportion decreasing from 27.25% in 1989 to 9.02% in 2018. The proportion of areas with an RSEI rating good decreased from 30.84% to 11.52% while the proportion of areas with bad rating increased from 4.73% to 19.11% during the study periods [82, 83].

**Fig 9:**
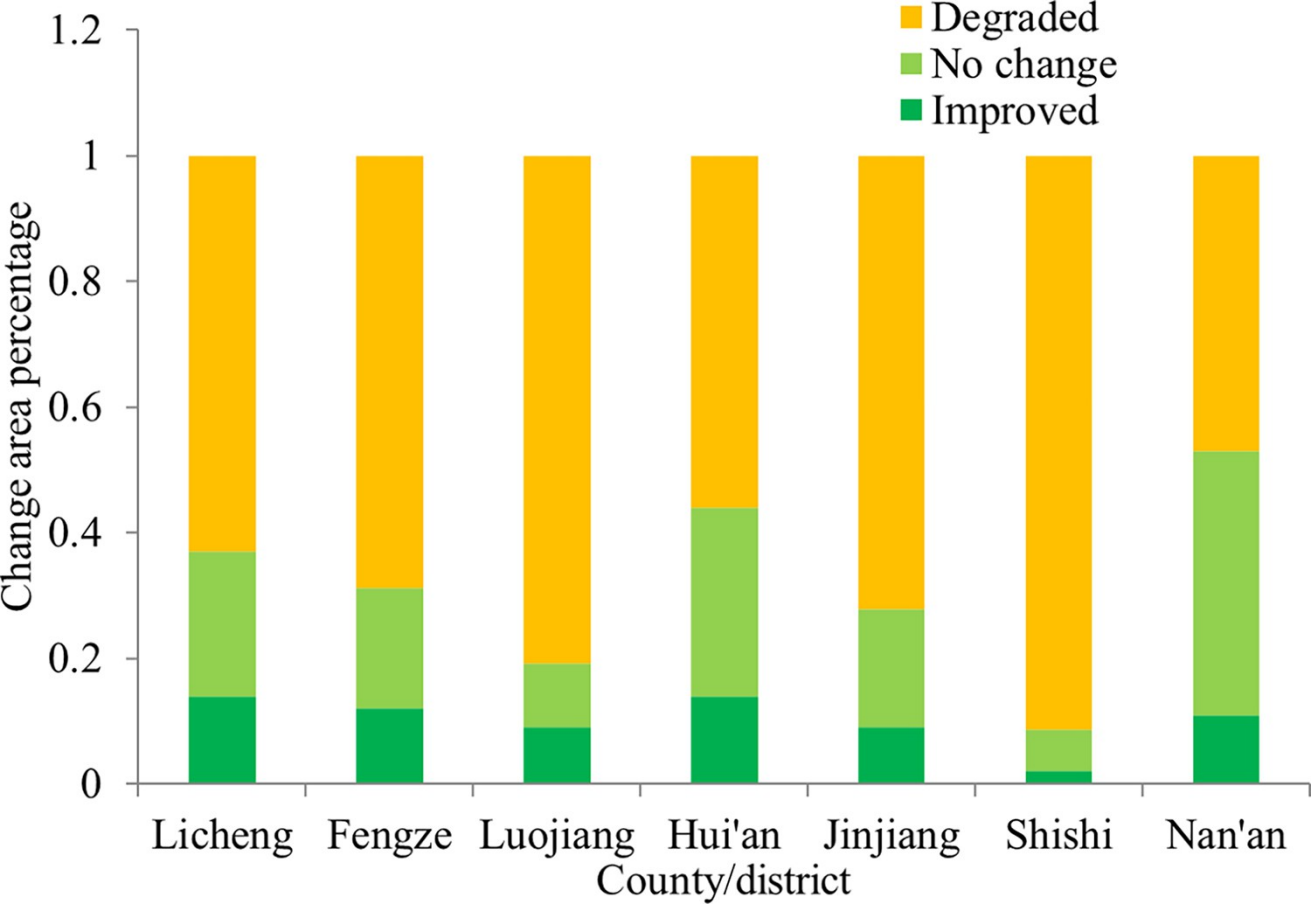
Ecological quality change in each county of Quanzhou during 1989–2018.

To compare the spatial and temporal changes in the ecological quality of Quanzhou during the period 1989 to 2018, the simple image differencing method is employed in the study [84]. Fig 9 shows the results of an analysis of changes in the ecological quality of 7 districts in Quanzhou. The proportion of improved areas was 10.16% and that of deteriorated areas was 68.44%, while 21.40% of study region areas remained unchanged. Evidently, the ecological condition of the main built-up land deteriorated while that of the mountains remained unchanged. In the districts of Licheng, Fengze, Luojiang, Hui’an, Jinjiang, Shishi and Nan’an, the coverage of deteriorated areas all exceeded that of the improved areas. These results indicate a decline in the ecological quality of Quanzhou between 1989 and 2018. Notably, the proportion of deteriorated areas is 91.33%, while only 2% of the study region areas improved in Shishi. Because of the urban expansion, more areas evidenced declining ecological quality than improved quality in all districts. The deterioration in the ecological quality in Quanzhou appears to be closely related to the large-scale expansion of cities and deforestation in these areas.

#### 3.4.2 Contribution rate of land use types to ecological quality change

In order to better the contribution of different land use types to a certain remote sensing ecological quality level, the study proposes a remote sensing ecological quality contribution rate index, which can more clearly show the effect of different land use types on ecological quality. The calculation method is as followed:

$$EQCT_{xy}=\frac{A_{xy}}{A_{y}}\times 100\%$$ (17)

Where *EQCT*_*XY*_ is the contribution rate index of different land use type to ecological quality, *A*_*XY*_ is the area occupied by the land use type *x* in the level *y* of ecological quality, *A*_*y*_ is the total area of level *y* of ecological quality.

**Fig 10:**
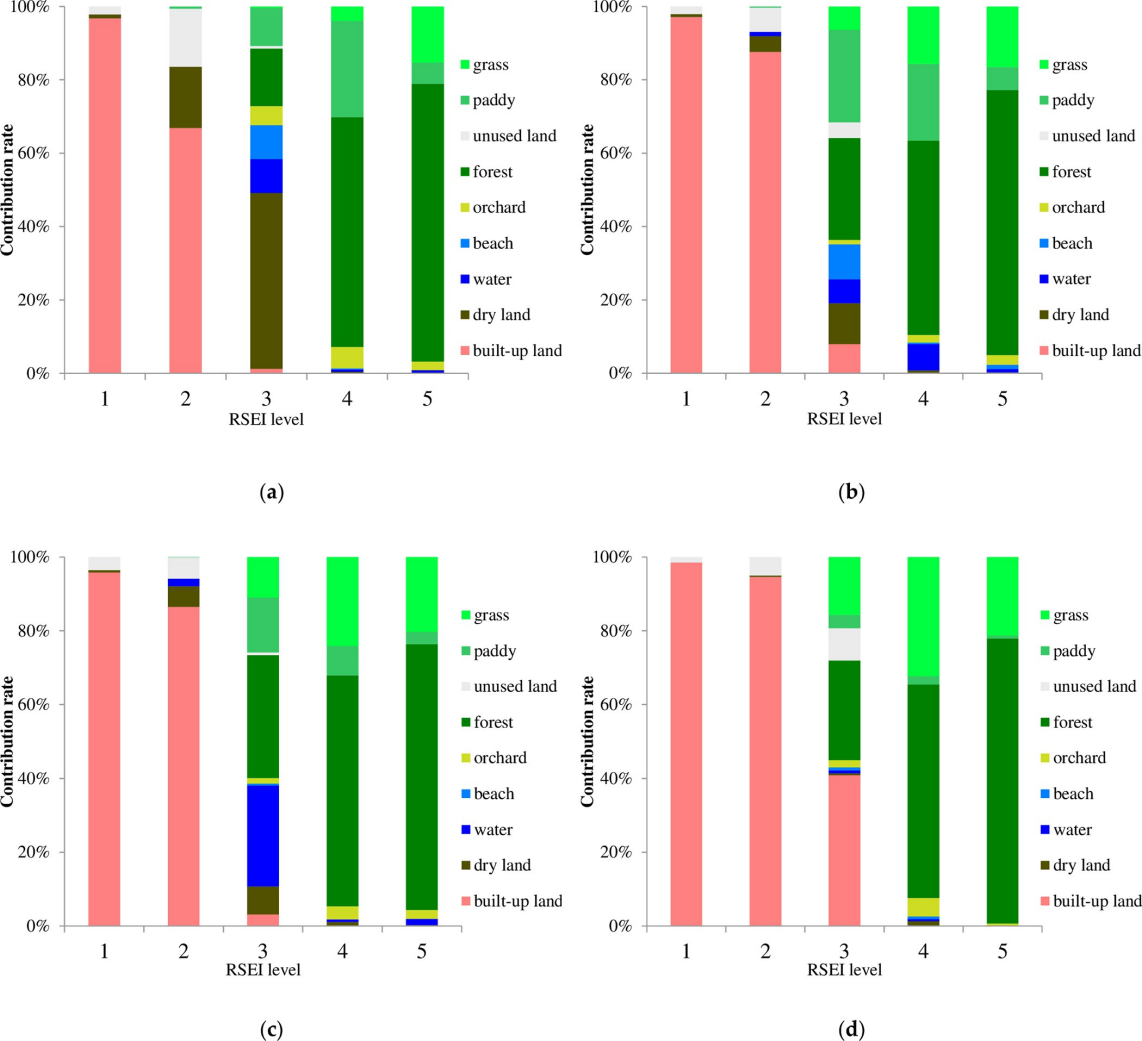
Contribution rate of various land use types to ecological quality in Quanzhou. (a): 1989. (b) 1999. (c) 2007. (d) 2018.

From Fig 10, it can be seen that the contribution of different land use types to urban remote sensing ecological quality is various. Most of the land types in the area with good ecological quality (Level 4 and Level 5) are forest, grass and arable land. The area with bad ecological quality (Level 1 and Level 2) is mainly built-up land and unused land, which indicates that forest, grass and arable land have the function of optimizing the ecology, while the built-up land and unused land have the function of deteriorating the ecology. Moreover, the contribution of different land use types to the same level of remote sensing ecological quality is not consistent in different years. The contribution rates of forest, grass and arable land to ecological quality are relatively stable, and the contribution rate of built-up land to the ecological quality has changed the most. Among them, the contribution rate of built-up land to the bad ecological quality (level 2) deterioration has increased from 66.88% in 1989 to 94.63% in 2018, which is due to the continuous expansion of construction land area and the decline of urban ecological quality in built-up land. With the improvement of people’s ecological awareness and effective management, it is gratifying that the contribution rate of newly-added built-up land to the acceptable ecological quality (level 3) increases accordingly in 2018.

## Discussion

**Fig 11:**
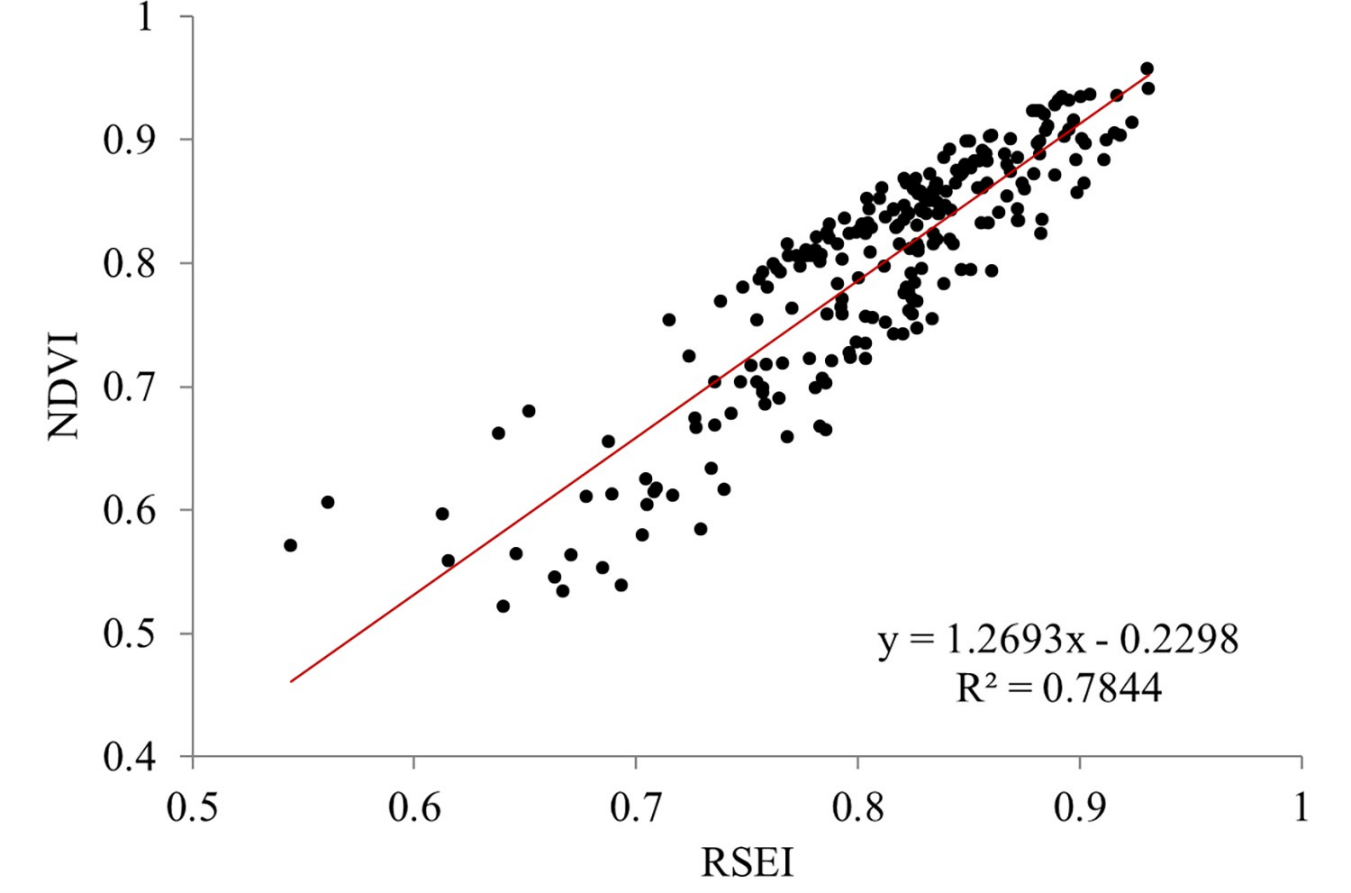
Relationship between RSEI and NDVI.

The spatial and temporal changes of a region’s ecological status are influenced by various complex factors. The LULC change entails a greater emphasis on the ecological nature of urbanization and is closely associated with the areas of built-up and cultivated land as well as green coverage. The spatial and temporal changes of construction land and vegetation have the same trend as the changes of ecological quality [85]. In order to quantitatively reveal the relationship between them, a regression analysis of the changes is carried out. Fig 11 shows that the result has a positive correlation (R^2^ = 0.7844) in the linear regression model. The slope value of 1.2693 is greater than 0, indicating an upward trend for RSEI with the increase of NDVI.

Quanzhou city is located on/near the river flood plain of the Jinjiang River and this is also the most productive field that has led to a substantial reduction in agricultural potential in the areas during the period of urbanization. Evidently, the area of arable land in Quanzhou declined sharply from 135.24 km^2^ in 1989 to 24.02 km^2^ in 2018. The decrease in the area of cultivated land is indicative of a decrease in NDVI value, which inevitably corresponds to a decrease in the RSEI value.

**Fig 12:**
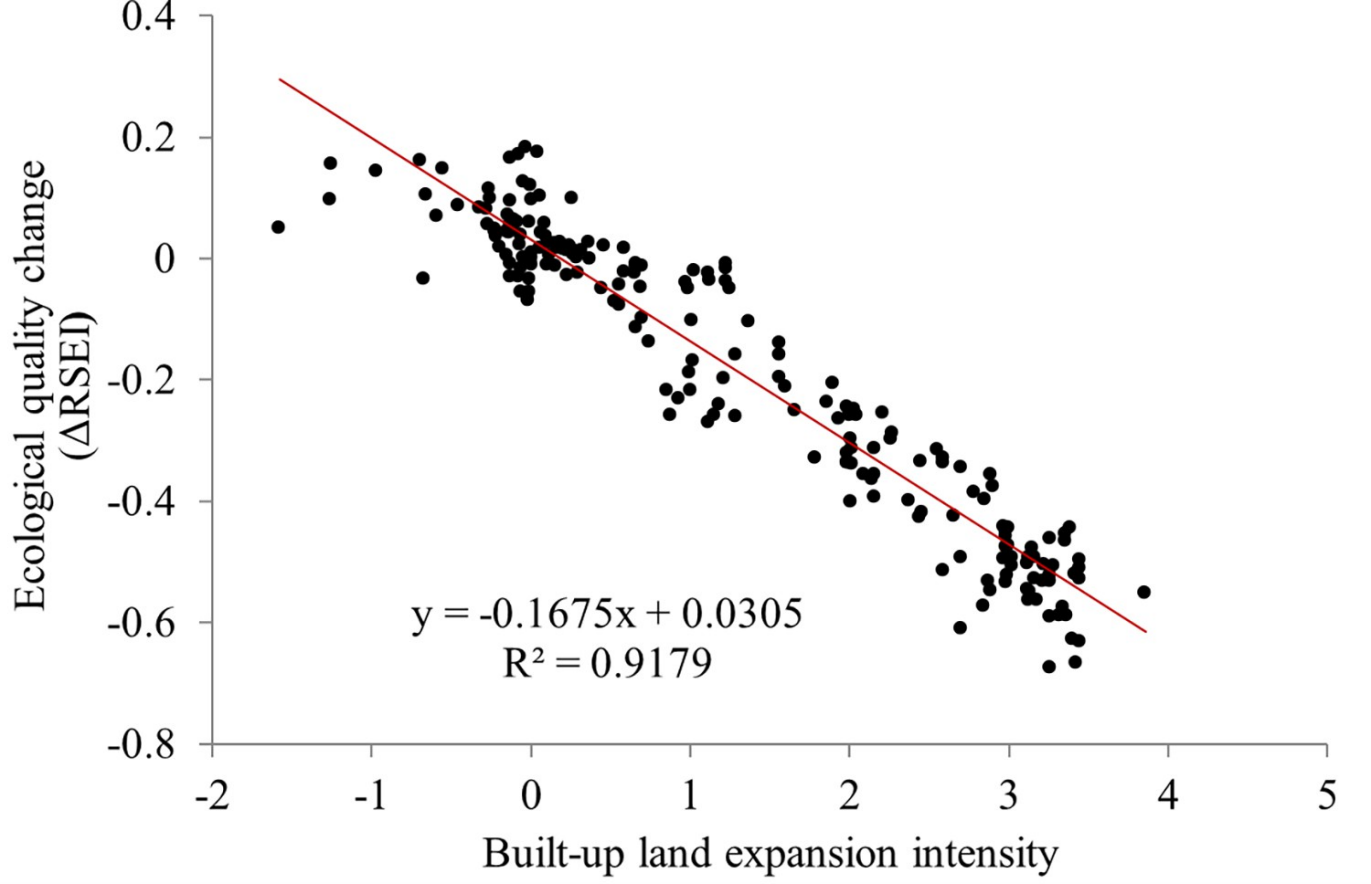
Relationship between built-up land expansion intensity and ecological quality change.

The driving forces for the above major land-use changes in Quanzhou city are believed to be over-fast economic growth. The economic growth was at the expense of the massive reduction of the valuable arable land and a rapid increase of the built-up land. The regression result (Fig 12) shows that RSEI and the built-up land expansion intensity have a significant linear negative correlation (R^2^ = 0.9179). As the built-up land expansion intensity increases, the ecological quality declines sharply. The increasing built-up land expansion intensity reflects a corresponding increase in the area of impervious surfaces, such as buildings and factories.

Land use/cover change is very important to ecological environment change, but many scholars often directly use remote sensing methods to extract ecological factors to study changes in the study of ecological environment [36–38, 60–61], without linking with land use change, which is not conducive to fundamentally analyze the driving factors of ecological environment change. As we all know, it is a very complex interactive coupling relationship between LULC, urbanization and ecological quality change, urbanization development is a process of human transformation of the natural environment, which will inevitably lead to huge changes in LULC, which have a huge impact on the ecological environment. Of course, this effect of LULC on ecology can be good or bad. In the primary stage of urbanization, due to the low socio-economic level, people’s ecological awareness is not high, and more attention is paid to how to quickly acquire natural resources and increase social wealth, such as building factories, building roads, resulting in the deterioration of the natural ecological environment. When urbanization develops to a relatively mature stage and the social economy reaches a certain scale, people’s awareness of ecological environment protection will be greatly improved. Since the advancement of various political policies has a profound impact on regional ecological quality, and this impact is complex, long-term and difficult to quantify [86, 87]. Based on various policy of the land use, ecology and socio-economic statistics of Quanzhou city, combined with the land use change and ecological quality change in the same period, this study comprehensively analyze the intrinsic influences and drivers of LULC and ecological quality of Quanzhou city by the multi-dimensional scaling analysis method [88].

**Fig 13:**
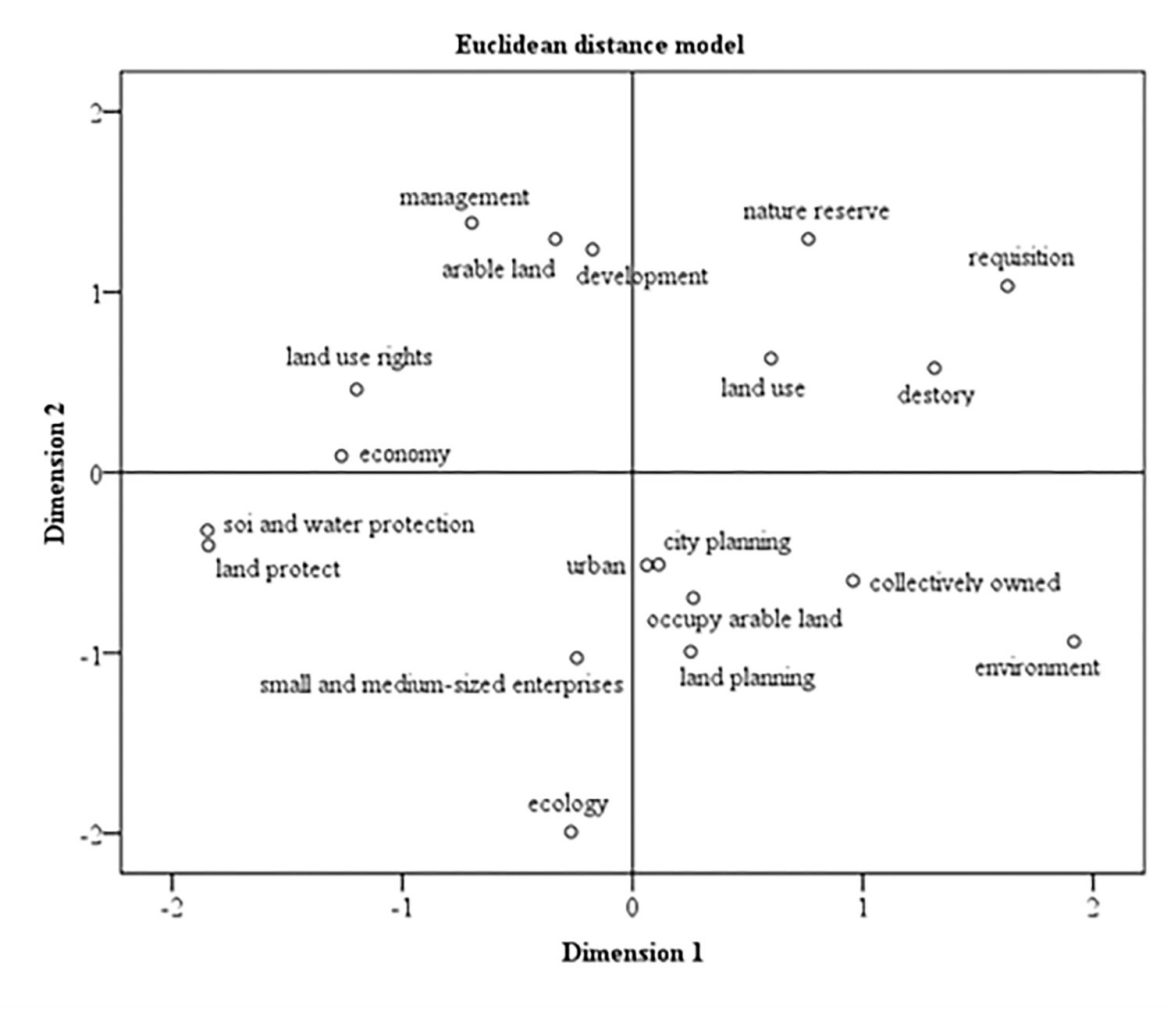
Multidimensional scaling map during 1989–2018.

From the multi-dimensional scale map from 1989 to 2018 (Fig 13), it can be seen that the core issues that government departments paid attention to included urban development, urban planning, land planning, and small and medium-sized enterprises to promote economic and social development during China’s reform periods. The main means of land management are land protection, collective ownership and water protection. In the early stage of the study period, the government is mainly committed to promoting the rapid development of the social economy, thereby rapidly promoting urban development. Since 2012, the government attaches equal importance to ecology and economic development, and policies and regulations on ecological protection, such as soil and water protection, and land protection, have been designated and gradually implemented.

The rate of urban/town spatial expansion always changes with the fluctuation of economic development. The Gross Domestic Product (GDP) was 5.348 billion yuan (RMB) in 1989 and greatly increased to 846.798 billion yuan in 2018. With the rapid development of the economy, the industry structure of Quanzhou city has changed significantly, which would demand more land for the expanding industry. Consequently, several industrial estates have been established during the study periods. Fast economic development also brings up increases in investment in housing construction, which leads the urban/town space to expand at an accelerated rate. The fast development of real estate resulted in the demand for land and thus the expansion of the built-up land use.

The significant economic activities also triggered deforestation, and urban expansion and building construction all demanded a large amount of accessible land with convenient transportation conditions. However, severe forest and arable land loss had a significant impact on the city’s ecological quality and sustainable development.

**Fig 14:**
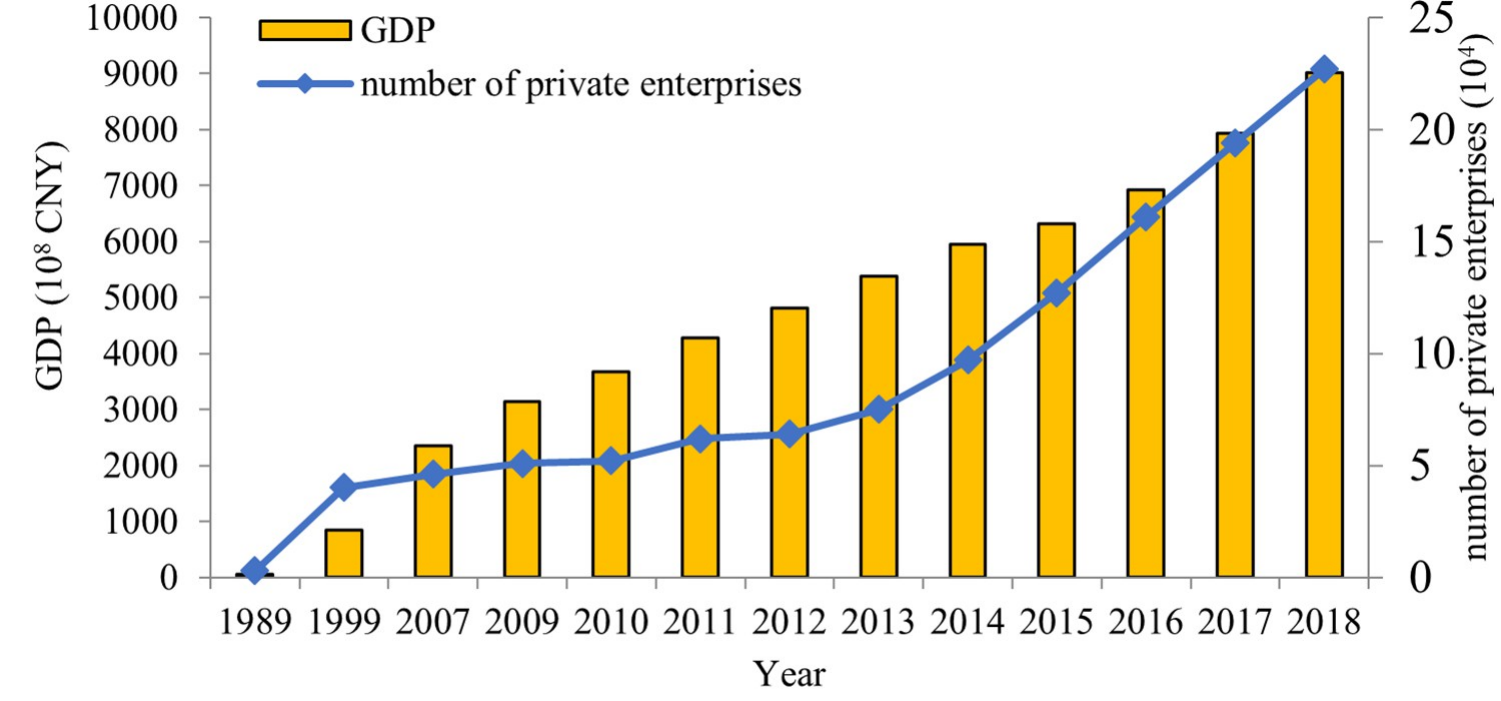
Change of GDP and the number of private enterprises of Quanzhou.

To further study, it had shown that satellite observation of LULC change was related to underlying socio-economic trends and the outcome of local policies [89]. As we all know, Quanzhou is one of the famous cities with the most developed private economy in China. According to the economic and social statistics released by the Quanzhou Municipal Bureau of Statistics, the market economy has developed rapidly from the reform and opening up. The number of private enterprises has increased significantly from 8,300 in 1989 to 141,800 in 2018, an increase of 17.08 times (Fig 14). The proportion of private enterprises in the total number of enterprises increased from 43.3% in 1989 to 92.6% in 2018. At present, the economic structure of Quanzhou has been formed mainly by food, textile and clothing, footwear, building materials and machinery industries, and the output of building materials and footwear account for 60% and 40% of the country, respectively. Chendi town in Jinjiang, known as “China Shoe Capital”, has more than 3000 footwear companies in the land of less than 40 km^2^.

With the rapid development of economics and increase of private enterprises, more and more land areas were used to satisfy the needs of people, resulting in lots of urban surfaces instead of croplands and forests, the evolution of the urban thermal environment pattern in Quanzhou was greatly affected by policy intervention, which was consistent with the research conclusion of Yang et al (2019) [90]. To seek high-income benefits drive people to plant high-value-added crops to replace traditional rice and wheat, which further impulses the LULC change of Quanzhou. Since the beginning of the 21st century, a large number of private enterprises have emerged in Jinjiang, Shishi and Fengze, which has led to a rapid increase in built-up land. A large number of talents, funds and technologies are gathered in the new built-up land, such as Chendai and Chidian town in Jinjiang, Huada street in Fengze and Qianjiang town in Shishi, and promote these areas to become new urban centers.

However, the significant change of LULC has greatly affected our existence and lives, and the rapid expansion of urban areas has a significant impact on regional ecological quality. To maintain the persistent development of economics and society, strict land-protected policies and logical land-used schemes should be put into practice all over the counties and cities, even in the whole nation. With the implementation of ecological and environmental protection policies, the deterioration of ecological quality has been curbed in Quanzhou. From the map of the contribution rate of various land use types to ecological quality in 2018, there are more acceptable ecological quality grades in the new urban land area, which also confirms this point. Obviously, the dynamics monitoring of LULC change through remote sensing technology can provide important information for us to discover and analyze the estate of LULC during the urbanization process. Moreover, the concept of ecology must be integrated to enable the healthy and sustainable development of the city and should be paid enough attention to take action in the decision-making and planning of urban economic development.

## Conclusion

In this study, Quanzhou, China, was selected as the study area. Based on Landsat remote sensing image data collected in Quanzhou from 1989 to 2018, the LULC were retrieved, the expansion intensity and expansion rate of urban and characteristics of the ecological quality were systematically depicted, and the driving factors of the variations were revealed. The following conclusions were obtained based on our systematical analysis.

1. Quanzhou city had undergone a dramatic change in LULC during the urbanization process from 1989 to 2018, which resulted in the loss of cropland and forest, thus drastically altering the land surface characteristics. The built-up land had increased sharply from 52.83 km^2^ in 1989 to 245.82 km^2^ in 2018, with an increased rate of 5.45% annually. Meanwhile, the arable land was lost 111.22 in the 29-year period, which was mostly converted to built-up land. The areas with the obvious expansion of built-up land were mainly distributed in Chendai Town and Xibin Town in the northeastern coastal zone, Zimao Town in the west and Lingyuan Street in the middle of Jinjiang, and Huada Street in the northeastern coastal zone of Fengze.
2. The rapid development of urbanization in Quanzhou has resulted in the large-scale expansion of construction land and destroyed the original natural ecology dominated by vegetation, which is the main reason for the decline in regional ecological quality. With the expansion of construction land, the ecological quality of Quanzhou city has declined significantly. The average RSEI has dropped by 70%. The area with poor ecological quality has increased by 142.03 km^2^, while the area with good ecological quality has decreased by 100.53 km^2^. The predominant RSEI rating was converted from good in 1989 to poor in 2018. The areas with low ecological quality were mainly located in the urban areas and towns of Licheng, Fengze, Jinjiang and Shishi, and the areas with good ecological quality are mainly located in the mountains of Licheng and Fengze.
3. In view of the analysis regarding the reasons for the observed changes in the Quanzhou ecological quality, government policies play a profound impact on land use changes, urbanization and ecological environment changes, and the spatial expansion of urban, the most developed private economy structures and the transfer of industrial centers were the important driving factors for the observed evolution of the ecological environment of these regions. The expansion of urban in Licheng, Fengze, Jinjiang and Shishi caused the overall decline in ecological quality, and the development of the private economy and transfer of industrial centers promoted these areas to become new urban centers. With the strengthening of the ecological and environmental protection policies of the government, the deterioration of ecological quality has been curbed in Quanzhou. Therefore, the concept of ecology and regional functions should be integrated scientifically to maintain a long and sustainable development during the process of urbanization.

## Supporting information

S1 File (ZIP)

## References

[1] Foley JA, Defries R, Asner GP, Barford C, Bonan G, Carpenter SR, et al. Global consequences of land use. Science 2005; 309(5734): 570–574. doi: 10.1126/science.1111772 16040698

[2] Mooney HA, Duraiappah A, Larigauderie A. Evolution of natural and social science interactions in global change research programs. Proc. Natl. Acad. Sci. U S A. 2013; 110: 3665–3672. doi: 10.1073/pnas.1107484110 23297237 PMC3586612

[3] McDonnell MJ, MacGregor-Fors I. The ecological future of cities. Science 2016; 352(6288): 936–938. doi: 10.1126/science.aaf3630 27199416

[4] Owen TW, Carlson TN, Gillies RR. An assessment of satellite remotely-sensed land cover parameters in quantitatively describing the climate effect of urbanization. Int. J. Remote Sens. 1998; 19(9): 1663–1681. 10.1080/014311698215171

[5] Carlson TN, Arthur ST. The impact of land use-land cover changes due to urbanization on surface microclimate and hydrology: a satellite perspective. Glob. Planet. Change 2000; 25(1–2): 49–56. 10.1016/S0921-8181(00)00021-7

[6] Zhang DH, Zhou CS, He BJ. Spatial and temporal heterogeneity of urban land area and PM_2.5_ concentration in China. Urban Climate. 2022; 45: 101268. 10.1016/j.uclim.2022.101268

[7] Zhou CS, Zhang DH, Cao YW, Wang YZ, Zhang GJ. Spatio-temporal evolution and factors of climate comfort for urban human settlements in the Guangdong—HongKong—Macau Greater Bay area. Front. Environ. Sci. 2022; 10: 1001064. 10.3389/fenvs.2022.1001064

[8] Vitousek PM, Mooney HA, Lubchenco J, Melillo JM. Human domination of Earth’s ecosystems. Science 1997; 277(5325): 494–499. 10.1126/science.277.5325.494

[9] Siedentop S, Fina S. Who sprawls most? Exploring the patterns of urban growth across 26 European countries. Environ. Plann. A 2012; 44(11): 2765–2784. 10.1068/a4580

[10] Kennedy RE, Andréfouët S, Cohen WB, Gómez C, Griffiths P, Hais M, et al. Bringing an ecological view of change to Landsat-based remote sensing. Front. Ecol. Environ. 2014; 12(6): 339–346. 10.1890/130066

[11] Lebed L, Qi J, Heilman P. An ecological assessment of pasturelands in the Balkhash area of Kazakhstan with remote sensing and models. Environ. Res. Lett. 2012; 7: 025203. 10.1088/1748-9326/7/2/025203

[12] Guerschman JP, Scarth PF, McVicar TR, Renzullo LJ, Malthus TJ, Stewart JB, et al. Assessing the effects of site heterogeneity and soil properties when unmixing photosynthetic vegetation, non-photosynthetic vegetation and bare soil fractions from Landsat and MODIS data. Remote Sens. Environ. 2015; 161: 12–26. 10.1016/j.rse.2015.01.021

[13] Zhang DH, Zhou CS, Xu WW. Spatial-temporal characteristics of primary and secondary educational resources for relocated children of migrant workers: the case of Liaoning province. Complexity. 2020; 2: 1–13. 10.1155/2020/7457109

[14] Yang J, Su J, Xia J, Jin C, Li X, Ge Q. The impact of spatial form of urban architecture on the urban thermal environment: A case study of the Zhongshan district, Dalian, China. IEEE Journal of Selected Topics in Applied Earth Observationsand Remote Sensing. 2018; 11(8): 2709–2716. 10.1109/JSTARS.2018.2808469

[15] Estoque RC, Murayama Y, Lasco RD, Mylnt SW, Pulhin FB, Wang CY, et al. Changes in landscape pattern of the La Mesa Watershed-The last ecological frontier of Metro Manila, Philippines. For. Ecol. Manag. 2018; 430: 280–290. 10.1016/j.foreco.2018.08.023

[16] Jaafari S, Sakieh Y, Shabani AA, Danehkar A, Nazarisamani AA. Landscape change assessment of reservation areas using remote sensing and landscape metrics (case study: Jajroud reservation, Iran). Environ. Dev. Sustain. 2016; 18: 1701–1717. 10.1007/s10668-015-9712-4

[17] Liang BQ, Weng QH. Assessing urban environmental quality change of Indianapolis, United States, by the remote sensing and GIS integration. IEEE J-STARS 2010; 4(1): 43–55. 10.1109/JSTARS.2010.2060316

[18] Zhang YS, Odeh IOA, Han CF. Bi-temporal characterization of land surface temperature in relation to impervious surface area, NDVI and NDBI, using a sub-pixel image analysis. Int. J. Appl. Earth Obs. Geoinf. 2009; 11(4): 256–264. 10.1016/j.jag.2009.03.001

[19] Levin SA. The problem of pattern and scale in ecology: The Robert H. MacArthur Award Lecture. Ecology 1992; 73(6): 1943–1967. 10.2307/1941447

[20] Pal S, Xueref-Remy I, Ammoura L, Chazette P, Gibert F, Royer P, et al. Spatio-temporal variability of the atmospheric boundary layer depth over the Paris agglomeration: An assessment of the impact of the urban heat island intensity. Atmos. Environ. 2012; 63: 261–275. 10.1016/j.atmosenv.2012.09.046

[21] Williams M, Longstaff B, Buchanan C, Llanso R, Dennison W. Development and evaluation of a spatially-explicit index of Chesapeake Bay health. Mar Pollut. Bull. 2009; 59(1–3): 14–25. doi: 10.1016/j.marpolbul.2008.11.018 19117579

[22] Willis KS. Remote sensing change detection for ecological monitoring in United States protected areas. Biol. Conserv. 2015; 182: 233–242. 10.1016/j.biocon.2014.12.006

[23] Zhang DH, Zhou CS, Sun DQ, Qian Y. The influence of the spatial pattern of urban road networks on the quality of business environments: the case of Dalian City. Environ. Dev. Sustain. 2022; 24: 9429–9446. 10.1007/s10668-021-01832-z

[24] Zhang DH, Zahng GJ, Zhou CS. Differences in Accessibility of Public Health Facilities in Hierarchical Municipalities and the Spatial Pattern Characteristics of Their Services in Doumen District, China. Land 2021; 10(11): 1249. 10.3390/land10111249

[25] Zhou CS, Zhang DH, He X. Transportation accessibility evaluation of educational institutions conducting field environmental education activities in ecological protection areas: A case Study of Zhuhai city. Sustainability 2021; 13(16), 9392. 10.3390/su13169392

[26] Kwok R. Ecology’s remote-sensing revolution. Nature 2018; 556: 137–138. doi: 10.1038/d41586-018-03924-9 29620755

[27] Reza MIH, Abdullah SA. Regional index of ecological integrity: A need for sustainable management of natural resources. Ecol. Indic. 2011; 11(2): 220–229. 10.1016/j.ecolind.2010.08.010

[28] Dubinin V, Svoray T, Dorman M, Perevolotsky A. Detecting biodiversity refugia using remotely sensed data. Landsc. Ecol. 2018; 33: 1815–1830. 10.1007/s10980-018-0705-1

[29] Mwaniki MW, Agutu NO, Mbaka JG, Ngigi TG, Waithaka EH. Landslide scar/soil erodibility mapping using Landsat TM/ETM+ bands 7 and 3 normalised difference index: A case study of central region of Kenya. Appl. Geogr. 2015; 64: 108–120. 10.1016/j.apgeog.2015.09.009

[30] Yang J, Sun J, Ge QS, Li XM. Assessing the impacts of urbanization-associated green space on urban land surface temperature: A case study of Dalian, China. Urban Forestry & Urban Greening. 2017; 22: 1–10. 10.1016/j.ufug.2017.01.002

[31] Li X, Zhou Y, Zhu Z, Liang L, Yu B, Cao W. Mapping annual urban dynamics (1985–2015) using time series of Landsat Data. Remote Sens. Environ. 2018; 216: 674–683. 10.1016/j.rse.2018.07.030

[32] Yang J, Zhan Y, Xiao X, Xia JC, Sun W, Li X. Investigating the diversity of land surface temperature characteristics in different scale cities based on local climate zones. Urban Climate. 2020; 34: 100700. 10.1016/j.uclim.2020.100700

[33] Estoque RC, Murayama Y. Monitoring surface urban heat island formation in a tropical mountain city using Landsat data (1987–2015). ISPRS J. Photogramm. Remote Sens. 2017; 133: 18–29. 10.1016/j.isprsjprs.2017.09.008

[34] Goward SN, Xue YK, Czajkowski KP. Evaluating land surface moisture conditions from the remotely sensed temperature/vegetation index measurements: An exploration with the simplified simple biosphere model. Remote Sens. Environ. 2002; 79(2–3): 225–242. 10.1016/S0034-4257(01)00275-9

[35] Coutts AM, Harris RJ, Phan T, Livesley SJ, Williams NSG, Tapper NJ. Thermal infrared remote sensing of urban heat: Hotspots, vegetation, and an assessment of techniques for use in urban planning. Remote Sens. Environ. 2016; 186: 637–651. 10.1016/j.rse.2016.09.007

[36] White DC, Lewis MM, Green G, Gotch TB. A generalizable NDVI-based wetland delineation indicator for remote monitoring of groundwater flows in the Australian Great Artesian Basin. Ecol. Indic. 2016; 60: 1309–1320. 10.1016/j.ecolind.2015.01.032

[37] Du PJ, Xia JS, Du Q, Luo Y, Tan K. Evaluation of the spatio-temporal pattern of urban ecological security using remote sensing and GIS. Int. J. Remote Sens. 2013; 34(3): 848–863. 10.1080/01431161.2012.714503

[38] Behling R, Bochow M, Foerster S, Roessner S, Kaufmann H. Automated GIS-based derivation of urban ecological indicators using hyperspectral remote sensing and height information. Ecol. Indic. 2015; 48: 218–234. 10.1016/j.ecolind.2014.08.003

[39] Yang J, Luo X, Jin C, Xiao X, Xia J (Cecilia). Spatiotemporal patterns of vegetation phenology along the urban–rural gradient in Coastal Dalian, China. Urban Forestry & Urban Greening. 2020; 54: 126784. 10.1016/j.ufug.2020.126784

[40] Xu HQ, Wang MY, Shi TT, Guan HD, Fang CY, Lin ZL. Prediction of ecological effects of potential population and impervious surface increases using a remote sensing based ecological index (RSEI). Ecol. Indic. 2018; 93: 730–740. 10.1016/j.ecolind.2018.05.055

[41] Xu HQ. Spatiotemporal dynamics of the bare soil cover in the Hetian basinal area of County Changting, China, during the past 35 years. Acta Ecologica Sinica 2013; 33(10): 2946–2953. 10.5846/stxb201204210575

[42] Hauser LT, Vu GN, Nguyen BA, et al. Uncovering the spatio-temporal dynamics of land cover change and fragmentation of mangroves in the Ca Mau peninsula, Vietnam using multi-temporal SPOT satellite imagery (2004–2013). Applied Geography. 2017, 86: 197–207. 10.1016/j.apgeog.2017.06.019

[43] Liu JY, Ning J, Kuang WH, et al. Spatio-temporal patterns and characteristics of land-use changein China during 2010–2015. Acta Geographica Sinica, 2018, 73(5): 789–802. 10.11821/dlxb201805001

[44] Yang J, Jin S, Xiao X, Jin C, Xia J (Cecilia), Li X, et al. Local climate zone ventilation and urban land surface temperatures: towards a performance-based and wind-sensitive planning proposal in megacities. Sustainable Cites and Society. 2019; 47:1–11. 10.1016/j.scs.2019.101487

[45] Song K, Wang YJ, Li Y. Monitoring of ecological environment changes in the Yangtze river economic belt (Jiangsu province) from 1999 to 2020 and analysis of the driving forces of human activities. Bulletin of Surveying and Mapping. 2021; 2: 7–12. 10.13474/j.cnki.11-2246.2021.0034

[46] Kong LL, Feng XF, Wu S, Liu ZC, Yao XC. Spatiotemporal dynamics and driving factor analysis of ecological quality change in the Lhasa urban circle from 1994 to 2017. Progress in Geography. 2022; 41(3): 437–450. 10.18306/dlkxjz.2022.03.007

[47] Xu HQ, Wang YF, Guan H, Shi TT, Hu XS. Detecting ecological changes with a remote sensing based ecological index (RSEI) produced time series and change vector analysis. Remote Sens. 2019; 11(20): 2345. 10.3390/rs11202345

[48] Dissanayake D, Morimoto T, Ranagalage M, Murayama Y. Land-use/land-cover changes and their impact on surface urban heat islands: case study of Kandy city, Sri Lanka. Climate 2019, 7(8): 250–270. 10.3390/cli7080099

[49] Liu JY, Buheaosier. Study on spatial-temporal feature of modern land-use change in China: using remote sensing techniques. Quat. Sci. 2000; 20(3): 229–239. 10.3321/j.issn:1001-7410.2000.03.003

[50] Liu SH, Wu CJ, Shen HQ. A GIS based Model of Urban Land Use Growth in Beijing. Acta Geographica Sinica 2000; 55(4): 407–416. 10.11821/xb200004003

[51] Xu HQ. A remote sensing urban ecological index and its application. Acta Ecol. Sin. 2013; 33: 7853–7862. 10.5846/stxb201208301223

[52] Huang C, Wylie B, Yang L, Homer C, Zylstra G. Derivation of a tasselled cap transformation based on Landsat 7 at-satellite reflectance. Int. J. Remote Sens. 2002; 23(8): 1741–1748. 10.1080/01431160110106113

[53] Baig MHA, Zhang LF, Shuai T, Tong QX. Derivation of a tasselled cap transformation based on Landsat 8 at-satellite reflectance. Remote Sens. Lett. 2014; 5: 423–431. 10.1080/2150704X.2014.915434

[54] Liu Q, Liu G, Huang C, Xie C. Comparison of tasselled cap transformations based on the selective bands of Landsat 8 OLI TOA reflectance images. Int. J. Remote Sens. 2015; 36(2): 417–441. 10.1080/01431161.2014.995274

[55] Estoque RC, Murayama Y, Myint SW. Effects of landscape composition and pattern on land surface temperature: An urban heat island study in the megacities of Southeast Asia. Sci. Total Environ. 2017; 577: 349–359. doi: 10.1016/j.scitotenv.2016.10.195 27832866

[56] De Araujo Barbosa CC, Atkinson PM, Dearing JA. Remote sensing of ecosystem services: A systematic review. Ecol. Indic. 2015; 52: 430–443. 10.1016/j.ecolind.2015.01.007

[57] Chander G, Markham BL, Helder DL. Summary of current radiometric calibration coefficients for Landsat MSS, TM, ETM+ and EO-1 ALI sensors. Remote Sens. Environ. 2009; 113(5): 893–903. 10.1016/j.rse.2009.01.007

[58] USGS. Landsat 8 Data Users Handbook. (2019-11-27). https://www.usgs.gov/landsat-missions/landsat-8-data-users-handbook

[59] Yang J, Guo A, Li Y, Zhang Y, Li X. Simulation of landscape spatial layout evolution in rural-urban fringe areas: a case study of Ganjingzi District. GIScience & Remote Sensing. 2019; 56(3): 388–405. 10.1080/15481603.2018.1533680

[60] Rhee J, Im J, Carbone GJ. Monitoring agricultural drought for arid and humid regions using multi-sensor remote sensing data. Remote Sens. Environ. 2010; 114(12): 2875–2887. 10.1016/j.rse.2010.07.005

[61] Weng Q, Fu P, Gao F. Generating daily land surface temperature at Landsat resolution by fusing Landsat and MODIS data. Remote Sens. Environ. 2014; 145: 55–67. 10.1016/j.rse.2014.02.003

[62] Xu HQ. Dynamic of soil exposure intensity and its effect on thermal environment change. Int. J. Climatol. 2014; 34: 902–910. 10.1002/joc.3738

[63] Lin L, Hao Z, Post CJ, Mikhailova EA, Yu K, Yang L, et al. Monitoring Land Cover Change on a Rapidly Urbanizing Island Using Google Earth Engine. Appl. Sci. 2020; 10(20): 7336. 10.3390/app10207336

[64] Xu HQ, Hu XJ, Huade G, He GJ. Development of a fine-scale discomfort index map and its application in measuring living environments using remotely-sensed thermal infrared imagery. Energy Build. 2017; 150: 598–607. 10.1016/j.enbuild.2017.06.003

[65] Nichol J. An emissivity modulation method for spatial enhancement of thermal satellite images in urban heat island analysis. Photogramm. Eng. Remote Sens. 2009; 5: 547–556. 10.14358/pers.75.5.547

[66] Simwanda M, Ranagalage M, Estoque RC, Murayama Y. Spatial analysis of surface urban heat islands in four rapidly growing African cities. Remote Sens. 2019; 11(14): 1645. 10.3390/rs11141645

[67] Jiménez-Muoz JC, Sobrino JA, Skokovic D, Mattar C, Cristóbal J. Land surface temperature retrieval methods from Landsat-8 thermal infrared sensor data. IEEE Geosci. Remote Sens. Lett. 2014; 11(10): 1840–1843. 10.1109/LGRS.2014.2312032

[68] Xu HQ. A new index for delineating built-up land features in satellite imagery. Int. J. Remote Sens. 2008; 29(14): 4269–4276. 10.1080/01431160802039957

[69] Rikimaru A, Roy PS, Miyatake S. Tropical forest cover density mapping. Trop. Ecol. 2002; 43: 39–47.

[70] Xu HQ, Shi TT, Wang MY, Lin ZL. Land cove changes in the Xiong’an new area and a prediction of ecological response to forthcoming regional planning. Acta Ecologica Sinica 2017; 37(19): 6289–6301. 10.5846/stxb201705210941

[71] Varshney A, Rajesh E. A comparative study of built-up index approaches for automated extraction of built-up regions from remote sensing data. J. Indian Soc. Remote Sens. 2014; 42: 659–663. 10.1007/s12524-013-0333-9

[72] Hu X, Xu H. A new remote sensing index based on the pressure-state-response framework to assess regional ecological change. Environ. Sci. Pollut. Res. 2019; 26: 5381–5393. doi: 10.1007/s11356-018-3948-0 30607851

[73] Mensah F, Adanu SK, Adanu DK. Remote sensing and GIS based assessment of land degradation and implications for Ghana’s ecological zones. Environ. Pract. 2015; 17(1): 3–15. 10.1017/S1466046614000465

[74] Carpio OV, Fath BD. Assessing the environmental impacts of urban growth using land use /land cover, water quality and health Indicators: A case study of Arequipa, Peru. Amer. J. Environ. Sci. 2011; 7(2): 90–101. 10.3844/ajessp.2011.90.101

[75] Gonçalves J, Alves P, Pôças I, Marcos B, Sousa-Silva R, Lomba Â, et al. Exploring the spatiotemporal dynamics of habitat suitability to improve conservation management of a vulnerable plant species. Biodivers. Conserv. 2016; 25: 2867–2888. 10.1007/s10531-016-1206-7

[76] Polydoros A, Cartalis C. Use of Earth Observation based indices for the monitoring of built-up area features and dynamics in support of urban energy studies. Energy Build. 2015; 98: 92–99. 10.1016/j.enbuild.2014.09.060

[77] Zhang DH, Zhou CS, Zhou Y, Zikiya B. Spatiotemporal relationship characteristic of climate comfort of urban human settlement environment and population density in China. Front. Ecol. Evol. 2022; 10: 953725. 10.3389/fevo.2022.953725

[78] Lanari R, Zeni G, Manunta M, Guarina S, Sansosti P. An integrated SAR/GIS approach for investigating urban deformation phenomena: a case study of the city of Naples, Italy. Int. J. Remote Sens. 2010; 25(14): 2855–2867. 10.1080/01431160310001647750

[79] Alcaraz-Segura D, Lomba A, Sousa-Silva R, Nieto-Lugilde D, Alves P, Georges D, et al. Potential of satellite-derived ecosystem functional attributes to anticipate species range shifts. Int. J. Appl. Earth Obs. Geoinformation 2017; 57: 86–92. 10.1016/j.jag.2016.12.009

[80] Acharya TD, Yang IT, Lee DH. Land cover classification using a KOMPSAT-3A multi-spectral satellite image. Appl. Sci. 2016; 6(11): 371. 10.3390/app6110371

[81] Yue H, Liu Y, Li Y, Lu Y. Eco-environmental quality assessment in China’s 35 major cities based on remote sensing ecological index. IEEE Access 2019; 7: 51295–51311. 10.1109/ACCESS.2019.2911627

[82] Xu HQ, Zhang H. Ecological response to urban expansion in an island city: Xiamen, Southeastern China. Sci. Geogr. Sin. 2015; 35(7): 867–872. 10.13249/j.cnki.sgs.2015.07.009

[83] Xu HQ, Ding F, Wen XL. Urban expansion and heat island dynamics in the Quanzhou region, China. IEEE J-STARS, 2009; 2(2): 74–79. 10.1109/JSTARS.2009.2023088

[84] Zhu Z. Change detection using Landsat time series: A review of frequencies, preprocessing, algorithms, and applications. ISPRS J. Photogramm. Remote Sens. 2017; 130: 370–384. 10.1016/j.isprsjprs.2017.06.013

[85] Chen W, Zhang J, Shi X, Liu S. Impacts of Building Features on the Cooling Effect of Vegetation in Community-Based MicroClimate: Recognition, Measurement and Simulation from a Case Study of Beijing. Int. J. Environ. Res. Public Health 2020; 17(23): 8915. doi: 10.3390/ijerph17238915 33266242 PMC7730582

[86] Delman J. Ecological civilization politics and governance in Hangzhou: new pathways to green urban development? Asia-Pacific Journal: Japan Focus. 2018; 16(17): 1–21.

[87] Hansen MH, Li H, Svarverud R. Ecological civilization: interpreting the Chinese past, projecting the global future. Global Environmental Change. 2018; 53: 195–203. 10.1016/j.gloenvcha.2018.09.014

[88] Xue ZH, Nie XY. Low-rank and sparse representation with adaptive neighborhood regularization for hyperspectral image classification. Journal of Geodesy and Geoinformation Science. 2022; 5(1): 73–90. 10.11947/j.JGGS.2022.0108

[89] Yang J, Wang Y, Xiu C, Xiao X, Xia J, Jin C. Optimizing local climate zones to mitigate urban heat island effect in human settlements. Journal of Cleaner Production. 2020; 275: 123767. 10.1016/j.jclepro.2020.123767

[90] Yang J, Wang Y, Xiao X, Jin C, Xia J (Cecilia), Li X. Spatial differentiation of urban wind and thermal environment in different grid size. Urban Climate. 2019; 28: 100458. 10.1016/j.uclim.2019.100458

